# Algae-Derived Bioactive Compounds as Platforms for Translational Biotechnology and Health Applications

**DOI:** 10.3390/biotech15020034

**Published:** 2026-05-15

**Authors:** Hannah Morris, Zoe Coombes, Zeinab El Dor, Valerie J. Rodrigues, Alla Silkina, Pietro Marchese, Mary Murphy, Jessica M. M. Adams, Frank Barry, Claudio Fuentes-Grünewald, Walid Rachidi, Deyarina Gonzalez

**Affiliations:** 1 Faculty of Medicine, Health and Life Science, Swansea University, Swansea SA2 8PP, UK; h.r.morris@swansea.ac.uk (H.M.); z.coombes@swansea.ac.uk (Z.C.); 2 Faculty of Pharmacy, Université Grenoble Alpes, 38000 Grenoble, France; zeinab.eldor@cea.fr (Z.E.D.); walid.rachidi@univ-grenoble-alpes.fr (W.R.); 3 Institute of Biological, Environmental and Rural Sciences (IBERS), Aberystwyth University, Gogerddan, Aberystwyth SY23 3EE, UK; var3@aber.ac.uk (V.J.R.); jaa@aber.ac.uk (J.M.M.A.); 4 Algal Research Laboratory, Green Economy Center BioHUB, Bioscience Department, Faculty of Science and Engineering, Swansea University, Swansea SA1 8EN, UK; a.silkina@swansea.ac.uk; 5 The Regenerative Medicine Institute, University of Galway, H91 TK33 Galway, Ireland; 0103205s@universityofgalway.ie (P.M.); mary.murphy@universityofgalway.ie (M.M.); frank.barry@universityofgalway.ie (F.B.); 6 Beacon Development Department, King Abdullah University of Science and Technology (KAUST), Thuwal 23955, Saudi Arabia; claudio.grunewald@kaust.edu.sa

**Keywords:** marine biodiscovery, marine bioactive compounds, microalgae, macroalgae, regenerative medicine, anti-cancer mechanisms, marine metabolites, cosmeceuticals

## Abstract

Marine macroalgae, microalgae, and associated microorganisms are increasingly recognised as valuable sources of bioactive compounds with applications across biotechnology and health. The environmental and ecological conditions they inhabit shape their metabolite diversity, leading to the production of high-value compounds such as sulphated polysaccharides, lipids, pigments, phenolics, and peptides. These compounds exhibit conserved biological activities that underpin potent antioxidant, anti-inflammatory, cytotoxic, and pro-regenerative effects with strong potential for translation. Although external factors drive rich metabolite diversity, continual variation can also lead to translational constraints including heavy-metal accumulation, inconsistency in extract composition, and regulatory complexity. This review examines the environmental drivers of metabolite diversity and the functional potential of bioactives derived from marine algae. We focus on their translational application within four areas of growing interest: nutraceuticals, cosmetics, regenerative medicine, and oncology, where emerging evidence suggests their promise as next-generation bioactive ingredients and therapeutic leads. In addition, insights from Irish and Welsh Small and Medium Enterprises (SMEs) are collated to identify key bottlenecks in commercialisation and the requirements for effective marine biodiscovery pipelines. We consider the importance of controlled cultivation, standardised analytics, preclinical testing platforms, and collaborative innovation ecosystems and highlight the need for coordinated scientific, technical, and regulatory advances to unlock the full translational potential of marine-derived compounds.

## 1. Introduction

Marine ecosystems cover approximately 70% of the Earth’s surface and host one of the most diverse and highly adaptive collections of organisms on the planet, forming a major reservoir of novel bioactive molecules with significant cosmetic, pharmaceutical, nutraceutical, and biotechnological potential [1]. Marine biodiscovery efforts are increasingly focused on microorganisms and primary producers—particularly microalgae (including cyanobacteria), macroalgae (seaweeds), marine bacteria and fungi. These groups exhibit unique metabolic capabilities shaped by long-term adaptation to fluctuating environmental pressures such as light intensity, temperature variation, salinity gradients, and biotic interactions [2,3]. These adaptive pressures drive the synthesis of structurally diverse metabolites, including pigments, polysaccharides, fatty acids, sterols, peptides, and secondary metabolites, many of which have already demonstrated relevance to human health and biotechnology [4,5,6].

Microalgae are unicellular photosynthetic organisms that inhabit a wide range of marine and brackish environments, contributing extensively to global primary productivity while producing high-value biomolecules with therapeutic, nutritional, and industrial potential [4,6,7]. Macroalgae similarly experience persistent abiotic and biotic stressors such as fluctuating UV exposure, temperature shifts, salinity changes, herbivory, and pathogenic attack. These stressors shape their metabolite profiles and promote the evolution of chemical defence molecules, photoprotective pigments, and antioxidant systems [8]. The ecological functions of these metabolites frequently align with human application areas, providing a translational bridge between marine chemical ecology and biotechnology.

Reflecting this emerging landscape, we highlight four major domains where marine-derived bioactives offer particularly strong translational potential: nutraceuticals and food security, skin biology and cosmetics, regenerative medicine, and anticancer therapeutics. These domains represent high-growth sectors with clear unmet needs that marine metabolites are well positioned to address due to their structural novelty, multi-target modes of action, and potential scalability [2,7,9]. Each domain is receiving increasing attention as biotechnological advances improve compound isolation, characterisation, and functional evaluation, enabling more direct alignment between discovery and end use.

**Figure 1:**
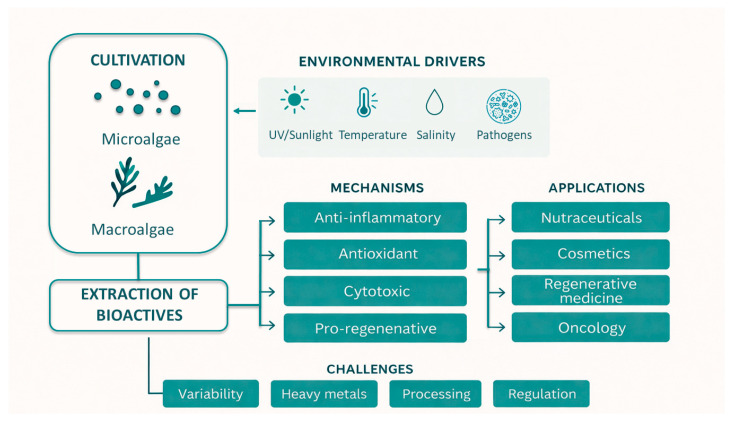
Schematic overview of marine algal bioactive discovery and application. Environmental drivers influence metabolite production in micro- and macroalgae, yielding bioactive compounds with diverse functional activities. These compounds are linked to applications in nutraceuticals, cosmetics, regenerative medicine, and oncology, with translation influenced by variability, processing, and regulatory challenges.

A key strength of marine biodiscovery lies in its potential sustainability. Microalgal biomass, for example, can be produced in controlled cultivation systems that minimise environmental footprint while enabling the optimisation of specific metabolite pathways [10,11,12]. Macroalgal harvesting and aquaculture likewise can support the renewable, low-impact and regenerative sourcing of bioactive compounds [13,14,15]. The intersection of sustainability, biochemical diversity, and translational relevance is increasingly framed within circular, blue- and bio-economy approaches, catalysing a shift from purely exploratory natural-product research toward application-driven biodiscovery in which mechanistic understanding is tightly coupled with real-world deployment and full-value utilisation of marine biomass [11,14,16] (Figure 1).

We aim to build on this foundation by:

1. Identifying the major algae-related sources of bioactive compounds;
2. Discussing the ecological and biochemical drivers of metabolite diversity;
3. Reviewing the principal functional classes of marine algae-derived bioactives;
4. Mapping the relevance of the principle functional classes to the four translational domains outlined above.

This integrated review combines ecological, biochemical, and application-focused perspectives, positioning marine biodiscovery as a versatile and sustainable platform capable of generating impactful innovations across health, nutrition, regenerative medicine, and oncology.

## 2. Marine Biodiscovery and Bioactive Sources

In their natural environment, marine macro- and microalgae are continually exposed to a wide spectrum of fluctuating abiotic pressures, including shifts in light intensity, oxidative temperature and salinity stress [4], and biotic challenges such as grazing by marine herbivores and pathogenic attacks. These environmental pressures, ecological interactions, and evolutionary metabolic adaptations collectively influence their distinctive metabolic profiles [5], stimulating the synthesis of protective and regulatory metabolites, such as pigments, fatty acids, protective exopolysaccharides and others, many of which exhibit bioactive properties relevant to human health and biotechnology [6].

### 2.1. Drivers of Bioactive Compound Diversity

#### 2.1.1. Light and Ultraviolet (UV) Radiation

Several key classes of metabolites in macroalgae are influenced by light, primarily those involved in photosynthesis, photoprotection and stress tolerance such as photosynthetic pigments and phenolic compounds. The wavelength of visible light is known to influence the production of photosynthetic pigments [17], with changes in light intensity or spectrum frequency altering the metabolic activity in aquaculture systems, while environmental light availability significantly influences the synthesis of phenolic compounds involved in antioxidant and protective functions [8]. Extracts from macroalgae exposed to high UV-light levels have been shown to demonstrate higher antioxidant activity compared to those obtained under lower exposure conditions, through either a higher phenolic content or a synergistic effect between phenolics and other metabolites [18]. Photosynthetic pigments from macroalgae, primarily carotenoids such as fucoxanthin and astaxanthin; phycobiliproteins; and chlorophyll derivatives, have demonstrated strong antioxidant, anti-inflammatory, neuroprotective, anticancer, antimicrobial, and photoprotective activities [9,19].

Several key classes of metabolites in microalgae are similarly influenced by light, particularly those associated with photosynthesis, photoprotection, and cellular stress responses, including photosynthetic pigments and phenolic compounds. The wavelength and intensity of visible light strongly regulate pigment biosynthesis in microalgae, with the spectral quality (e.g., blue, red, or green light) affecting the accumulation of chlorophylls, carotenoids, and phycobiliproteins through the modulation of photosynthetic efficiency and light-harvesting systems. Variations in light intensity and photoperiod can significantly alter metabolic fluxes in microalgal cultures, impacting both biomass productivity and the synthesis of high-value metabolites [12].

Light availability also plays a crucial role in the production of phenolic compounds and other antioxidant molecules in microalgae, which are involved in protection against oxidative stress. Exposure to elevated light intensities or UV radiation induces the generation of reactive oxygen species (ROS), triggering adaptive responses that enhance the accumulation of antioxidant compounds, including phenolics, carotenoids (e.g., β-carotene, lutein, astaxanthin), and mycosporine-like amino acids (MAAs) [20]. Similarly to macroalgae, increased UV exposure in microalgae has been associated with enhanced antioxidant activity of extracts, either due to higher phenolic content or synergistic interactions between multiple bioactive compounds.

Photosynthetic pigments in microalgae—including chlorophylls (a and b), carotenoids (such as β-carotene, lutein, and astaxanthin), and phycobiliproteins (e.g., phycocyanin and phycoerythrin)—are widely recognised for their diverse bioactivities [21]. These compounds exhibit strong antioxidant, anti-inflammatory, anticancer, antimicrobial, neuroprotective, and photoprotective properties, making microalgae a highly promising and scalable source of functional ingredients for applications in nutraceuticals, pharmaceuticals, cosmetics, and food systems.

#### 2.1.2. Temperature Fluctuations

In intertidal macroalgae, temperature is an abiotic stressor that activates chemical defence pathways and stimulates the production of secondary metabolites as adaptive responses to dynamic, and often harsh, environmental conditions [22]. In a study on the green macroalgae *Halimeda macroloba*, key physiological and biochemical pathways were reshaped by altered temperature conditions. In sub-optimal 25 °C conditions, reduced photosynthetic performance was accompanied by increases in oleic acid to maintain membrane fluidity and a significant accumulation of selenocysteine, selenomethionine, and related selenoproteins to help counteract low-temperature-induced oxidative stress. In optimal 28 °C conditions, photosynthetic efficiency, calcium content, and general metabolic performance peak, reflecting a stable metabolic state. In contrast, 31 °C triggers heat-responsive shifts, including the upregulation of proteins involved in photosynthesis and an accumulation of mannitol, acting as an antioxidant against environmental stress [23]. In subtidal kelps, mannitol levels also rise markedly during the summer months [24], though in deeper waters the temperature change is less marked and this is more likely due to the to increased light length and intensity during this period.

Extremophile microalgae such as the red algae *Galdieria sulphuraria* and *Cyanidioschyzon merolae* have received increasing scientific and industrial/commercial attention due to their poly-extreme natural adaptation to high temperatures > 45 °C and low pH levels < 4, making them particularly ideal candidates for industrial applications where pH and temperature stability in different processes is required [10,25].

#### 2.1.3. Salinity Changes

Salinity can influence metabolite production in macroalgae, particularly in relation to phenolic compounds. Most studies on Phaeophyceae report salinity shifts either suppressing or enhancing phenolic levels depending on species tolerance: tolerant Phaeophyceae maintain high phenolic content under hypersaline conditions, while others show reduced phenolics and antioxidant activity when exposed to elevated salinity [26]. Salinity has also been shown to strongly drive mannitol production in *Fucus vesiculosus*, with marine ecotypes containing more mannitol than those growing in lower salinity estuarine environments [27]. Furthermore, both ecotypes are able to adjust to reach the mannitol concentration of the other when placed in the alternative environment if light is also provided to enable photosynthesis and support long-term osmotic adjustment [27]. Additionally, decreases in the protein concentrations of red (*Gracilaria* spp.) and brown (*Sargassum* spp.) macroalgae have been associated with salinity increases [28].

Under increased salinity, microalgae maintain osmotic balance by accumulating compatible solutes (osmolytes) such as glycerol, glycine betaine, proline, and soluble sugars. A well-documented example is *Dunaliella salina*, which can accumulate high intracellular concentrations of glycerol (up to 50–60% of cell dry weight under hypersaline conditions), enabling survival in extreme salinity while maintaining photosynthetic activity [29]. This osmotic adjustment is often accompanied by significant shifts in carbon partitioning.

Salinity stress also enhances the accumulation of lipids, particularly neutral lipids (triacylglycerols, TAGs), as part of an energy storage and stress mitigation strategy. For instance, *Chlorella vulgaris* [30] and *Nannochloropisis salina* [31] have been shown to increase lipid content under elevated salinity, with reported increases of up to 1.5–2-fold depending on cultivation conditions. Similarly, salinity-induced oxidative stress promotes the synthesis of carotenoids, which play a protective role against reactive oxygen species (ROS). In *Dunaliella salina*, β-carotene accumulation can exceed 10% of dry biomass under high salinity and light stress [32].

#### 2.1.4. Grazing by Marine Herbivores

Macroalgae function within complex ecological networks where many of their secondary metabolites play key roles in defence, signalling, and interactions with other organisms. Herbivory from common marine grazers such as fish, sea urchins, amphipods, and molluscs has driven the evolution of diverse chemical defence strategies. These include the production of deterrent metabolites providing strong chemical unpalatability across Rhodophyta species, for example in *Plocamium* spp. which produce diverse mixtures of halogenated monoterpenes [33]. Halogenated monoterpenes have been known to exhibit significant biological activity such as anticancer, anti-plasmodial, and insecticidal qualities, with higher halogen proportions and their position within the monoterpene skeleton impacting their properties [34]. Within Phaeophyceae, a study on *Dictyota menstrualis* exhibited clear inducible chemical defences in response to grazing by the amphipod *Ampithoe longimana* through rapid increases in diterpenoids, resulting in reduced palatability and lower subsequent grazing, with concentrations often localised within damaged tissues. Diterpenoids exhibit a wide range of biological activities, functioning as potent antioxidants, demonstrating significant anti-inflammatory properties, and contributing to the modulation of immune responses [35].

#### 2.1.5. Microbial Pathogens

When exposed to bacteria, fungi, or competing algae through pathogenic attack, macroalgae synthesise diverse antimicrobial compounds including terpenoids, phenolics, polysaccharides, and halogenated metabolites which inhibit pathogen growth, damage cell walls, or disrupt membrane integrity [36]. Many of these compounds, such as sargafuran from Phaeophyceae *Sargassum macrocarpum*; or peyssonoic acids from Rhodophyta *Peyssonnelia* sp., act by lysing microbial cells or blocking key metabolic processes [36,37]. In Rhodophyta *Ceramium rubrum*, mixed extracts show strong antimicrobial activity compared to purified individual compounds, suggesting that macroalgae are employing complex chemical cocktails rather than single metabolites for defence [38].

Antifouling compounds range from fatty acids and terpenoids to alkaloids, pyrroles, and lipopeptides; inhibiting microbial attachment, disrupting quorum-sensing pathways, and preventing settlement of larger fouling organisms [39]. Additional metabolites such as phlorotannins, sulphated polysaccharides, carotenoids, and fatty acids can interfere with cell signalling, suppress biofilm formation, or disrupt extracellular polymeric substances, further enhancing resistance to colonisation [40]. Together, these chemical defences form a sophisticated antifouling system that contributes to the surface integrity and ecological success of marine macroalgae, with many potential applications across medicine, agriculture, food systems, aquaculture and environmental management.

Although not discussed at length in this review, it is important to highlight that marine microorganisms themselves similarly produce a wide spectrum of secondary metabolites that function as ecological defence molecules or signalling compounds. Collectively, marine microalgae and associated microorganisms contribute substantially to the diversity of marine-derived natural products currently being explored for biomedical applications [7]. For example, microalgal species *Porphyridium purpureum* produces sulphated polysaccharides that act as defence molecules, boasting antiviral and anti-inflammatory properties [12].

## 3. Functional Classes of Marine Bioactive Compounds

Both micro- and macroalgae produce numerous classes of bioactive molecules with essential roles in defence, signalling, metabolism, and maintaining cellular and structural integrity. Because these molecules frequently display potent biological activities, algae have emerged as rich reservoirs with demonstrated biomedical relevance [41].

### 3.1. Lipids and Lipid-Derived Mediators

#### 3.1.1. Lipids and Lipid-Derived Mediators in Macroalgae

Macroalgae possess highly distinctive lipid profiles comprising polyunsaturated fatty acids (PUFAs), PUFA derivatives, membrane-associated glycolipids, phospholipids and non-phosphorous glycerolipids [42]. Chlorophyta typically show lower levels of long-chain PUFAs compared with Rhodophyta and Phaeophyceae samples, which have relatively high levels of eicosapentaenoic acid (EPA); but all exhibit low n-6:n-3 ratios, indicating strong potential for use in health-promoting and sustainable nutritional applications [43].

Macroalgae also produce a rich suite of oxylipins and eicosanoids, oxygenated derivatives of polyunsaturated fatty acids that act as key chemical mediators in both defence and signalling. These compounds include prostaglandins, leukotrienes, and hydroxy and hydroperoxy fatty acids, many of which closely resemble the eicosanoids found in mammals [44]. Studies show that macroalgal oxylipins participate in stress signalling, innate immunity and chemical defence, often deterring herbivores, pathogens, or fouling organisms. They also display promising pharmacological activities, including anti-inflammatory and antimicrobial effects, with prostaglandins and hydroperoxyeicosatetraenoic acid being potentially suitable as valuable tools for drug discovery and biomedical research [45]. Glycolipids within macroalgae cell membranes include monogalactosyldiacylglycerides (MGDGs), digalactosyldiacylglycerides (DGDGs), and sulfolipids such as sulfoquinovosyldiacylglycerides (SQDGs), which all exhibit broad bioactivity, notably antiviral, anti-inflammatory, antibacterial, and antitumor effects [46].

Macroalgae are also rich in unique sterols, with fucosterol, a predominant sterol in Phaeophyceae, showing diverse bioactivities including antioxidant, antidiabetic, anti-inflammatory, neuroprotective, anti-obesity, and anticancer effects [47]. Due to this chemical diversity and the structural novelty of marine-derived lipids, macroalgal lipids are increasingly recognised as high-value targets in marine pharmacology, with significant potential for nutraceutical, pharmaceutical, and therapeutic development.

#### 3.1.2. Lipids and Lipid-Derived Mediators in Microalgae

Microalgae are important sources of polyunsaturated fatty acids (PUFAs), including eicosapentaenoic acid (EPA) and docosahexaenoic acid (DHA), which play essential roles in cardiovascular health, inflammation regulation, and neural development. EPA is abundantly produced by microalgae such as *Nannochloropsis oceanica* and *Phaeodactylum tricornutum*, while DHA is particularly associated with species such as *Schizochytrium* sp. and *Crypthecodinium cohnii* [48]. These fatty acids are widely used in nutraceutical formulations and infant nutrition products due to their established physiological benefits. In addition to structural lipids, microalgae produce lipid-derived signalling molecules such as oxylipins, which have demonstrated anti-inflammatory, antimicrobial, and immunomodulatory activities [49]. Exploring fatty acids, especially PUFAs, in new biotechnological microalgae platforms is important; the Dinoflagellate *Amphidinium carterae*, for example, has been identified as a promising source of different lipids with a range of applications in the pharmaceutical industry [50].

### 3.2. Polysaccharides and Glycoconjugates

#### 3.2.1. Polysaccharides and Glycoconjugates in Macroalgae

All macroalgae are prolific producers of structurally diverse polysaccharides that exhibit unique biological properties. These polysaccharides vary in monosaccharide composition, sulphation patterns, and branching, resulting in a wide range of functional and biomedical applications [51].

Alginate is found in Phaeophyceae and consists of a linear copolymer of β-D-mannuronic and α-L-guluronic acids that forms a hydrogel through ionic crosslinking with Ca^2+^, enabling broad biomedical use [52]. Reviews highlight alginate’s biocompatibility, biodegradability, and physicochemical properties, supporting applications in wound dressings, drug delivery, tissue engineering, and 3D bioprinting. Alginate-based nanofibers and nanoparticles further improve targeted delivery and regenerative applications, expanding its role as a versatile marine-derived biopolymer [53,54].

Sulphated polysaccharides exhibit important pharmacological properties including antioxidant, anticoagulant, anti-inflammatory, antiviral, and anticancer effects which are linked to their molecular weight, sulphate content, and sugar composition [55]. Several studies highlight the ability of these molecules to inhibit cancer cell growth and act similarly to heparin in anticoagulation [56,57,58]. In agriculture, macroalgal-derived polysaccharides function as sustainable biostimulants that enhance plant growth through enhanced establishment and yield and improve tolerance to abiotic stresses such as salinity by regulating nutrient uptake and acting as signalling molecules [59].

Fucoidan, a sulphated polysaccharide rich in L-fucose and sulphate groups, also contributes significant therapeutic potential. Its anticoagulant activity is strongly influenced by its molecular weight and degree of sulphation, with studies demonstrating potent heparin-like effects [60]. Fucoidan exhibits antiviral properties, including inhibition of HSV-1, and broader anti-inflammatory, anticancer, and immunomodulatory effects driven by structure–activity relationships tied to sulphate content and monosaccharide composition [61].

Carrageenans are linear sulphated galactans typically occurring in kappa, iota and lambda forms, each defined by distinct sulphation patterns that shape their gelling behaviour and broad functionality [51]. Carrageenan-based hydrogels show strong water absorption, negative charge, and readily modifiable functional groups, supporting their use in drug delivery, wound healing, and tissue engineering. Beyond these applications, carrageenans are widely employed in pharmaceutical formulations, environmental remediation, and bio-nanocomposites due to their gel-forming and stabilising capacities, with chemically modified and hybrid platforms further enhancing mechanical performance and biological functionality [62].

Specific Rhodophyta contain agar and its component agarose [63], which are derived from species such as *Gracilaria edulis* and serve as key gelling and stabilising agents with applications in microbial culturing, pharmaceuticals, and agriculture, while their bioactive derivatives exhibit antioxidant and other health-relevant properties [64].

Chlorophyta produce several bioactive polysaccharides, with ulvan, a sulphated heteropolysaccharide composed mainly of rhamnose, xylose, glucuronic acid, and iduronic acid, being the most prominent [65]. Ulvan exhibits a broad spectrum of antioxidant, anticancer, immunomodulatory, and antiviral activities, making it an increasingly popular candidate in biomedical research. Its structural resemblance to mammalian glycosaminoglycans enables effective integration into hybrid biomaterials for drug delivery and wound repair [66]. Beyond ulvan, green macroalgae also contain additional functional polysaccharides such as cellulose, xyloglucan, and glucuronan which contribute to additional prebiotic, structural, and bioactive properties. Together, these green-seaweed-derived polysaccharides are increasingly recognised for their potential in designing biocompatible, sustainable, and multifunctional biomaterials for future biomedical and biotechnological applications [66,67].

Macroalgae also produce a wide spectrum of bioactive glycoconjugates, including structurally diverse glycolipids such as monogalactosyldiacylglycerols, digalactosyldiacylglycerols, and sulfoquinovosyldiacylglycerols, which function as membrane-associated amphiphilic molecules with roles in antioxidant, antiviral, immunomodulatory, and pharmaceutical applications [46,68,69]. In addition, macroalgae contain glycoproteins, such as lectin and structural cell-wall glycoproteins, many of which exhibit antimicrobial, antihypertensive, anti-inflammatory, and other health-promoting activities [70,71]. Collectively, these glycolipid and glycoprotein glycoconjugates contribute to macroalgae’s growing value as a source of functional biomolecules with applications in nutrition, therapeutics, and biomaterial development.

#### 3.2.2. Polysaccharides and Glycoconjugates in Microalgae

As with macroalgae, extracellular polysaccharides and sulphated polysaccharides produced by marine microalgae have attracted attention due to their biological activities, including their antioxidant, antiviral, anticoagulant, and immunomodulatory effects [72].

Species such as *Porphyridium purpureum* produce sulphated exopolysaccharides rich in xylose, glucose, and galactose residues that exhibit antiviral activity against enveloped viruses and potential anti-inflammatory effects [21]. These polymers also possess rheological properties that make them attractive for pharmaceutical formulations, cosmetics, and biomedical materials. Other red microalgae, including *Porphyridium cruentum*, produce similar sulphated polysaccharides that have demonstrated anticoagulant activity comparable to heparin in some in vitro systems [73]. *Leptolyngbya* sp. produce bioactive extracellular polymers composed mainly of rhamnose, arabinose, mannose and glucose; all of these sugar monomers have shown significant antimicrobial activity against the fungi *Aspergillus niger* and anti-proliferative effects against cancer cells [74].

### 3.3. Pigments and Phenolic Compounds

#### 3.3.1. Pigments and Phenolic Compounds in Macroalgae

Pigmented metabolites from macroalgae have frequently been shown to exhibit anticancer, antioxidant and antibacterial properties, occurring as three main forms: chlorophylls, carotenoids and phycobiliproteins [34]. Chlorophylls are non-polar green pigments which play a pivotal role in photosynthesis, as sunlight capture or accessory pigments [75]. Carotenoids are red, orange or yellow; and act as accessory pigments which transport the energy to chlorophylls and protect against photooxidative damage [34]; and are classified into two depending on their molecular structure, with xanthophylls containing oxygen atoms in their structure and hydrocarbon carotenes without [76]. Within the xanthophylls, astaxanthin and fucoxanthin are significant molecules and these have been singled out for pharmacological interest. Being antioxidant, anticarcinogenic, anti-inflammatory and neuroprotective, they have the potential as candidates for chronic disease prevention or treatment, especially for neurodegenerative diseases [77] and ocular function [78]; fucoxanthin has also been identified as a functional food ingredient which can reduce diabetes and obesity risks [76].

Phycobiliproteins, appearing purple and blue [75], include phycoerythrin and phycocyanin, and constitute up to 8% dry weight of red algae composition. With their antioxidant and high absorbance properties they are widely used in biomedical and pharmaceutical sectors [76]. Collectively, with an interest in natural dyes and colours, these pigments have the potential to positively impact health whilst being used as colour additives.

Phlorotannins are found within vesicles in the cytoplasm of Phaeophyceae cells [79] and are oligomers or polymers of the phenol-based molecule phloroglucinol [80]. Though research on these molecules is still limited, several have been isolated, characterised and shown to exhibit potent antioxidant effects [79].

#### 3.3.2. Pigments and Phenolic Compounds in Microalgae

Marine microalgae synthesise a variety of pigments including carotenoids, chlorophyll derivatives, and phycobiliproteins, many of which exhibit strong antioxidant and photoprotective properties [81]. Astaxanthin, produced by *Haematococcus pluvialis*, is one of the most powerful known natural antioxidants and is widely used in nutraceutical and cosmetic applications. Fucoxanthin, abundant in diatoms such as *Phaeodactylum tricornutum* and *Odontella aurita*, has demonstrated anti-obesity, anti-inflammatory, and anticancer properties [82]. Cyanobacterial pigments such as phycocyanin, produced by *Arthrospira platensis* (Spirulina), exhibit antioxidant, hepatoprotective, and neuroprotective activities and are increasingly explored as natural therapeutic agents.

The new species *Monoraphidium* sp. produce a range of carotenoids including astaxanthin, violaxanthin, and lutein, among others; all of these pigments have well-known pharmaceutical potential as natural antioxidants, providing eye health effects or anticancer activity [83]. In the case of extremophile microalgae species such as *Galdieria* or *Cyanidioschyzon*, pigment extraction (especially phycobiliproteins) is of interest due to its simple and sustainable extraction process (combination of micro- and ultrafiltration using water as the main extractor carrier) and potential (in the case of *Phycocyanin*) use in industrial applications that require thermostable pigments [10].

### 3.4. Peptides and Secondary Metabolites

#### 3.4.1. Peptides and Secondary Metabolites in Macroalgae

The protein fractions within macroalgae differ by taxonomy, with Rhodophyta and Chlorophyta typically containing more protein (10.2–22.7 and 10.7–25.9%, respectively) than Phaeophyceae (1.1–26.8%), though large variation is seen for all types [52]. Within the category of proteins, macroalgae contain peptides, glycoproteins, mycosporine-like amino acids (MAAs), lectins, and enzymes, and within Rhodophyta, phycobiliproteins (as discussed in Section 3.3.1 on pigments).

Macroalgae bioactive peptides can be released using a range of processes including enzymic hydrolysis, acid, alkaline or hot water extractions, or fermentation—though enzymic hydrolysis is the most common [84]. Through hydrolysis, peptides 3–20 amino acids in length with bioactive properties are produced, showing anticancer, antioxidant and antithrombosis properties [79], with their biofunctionalities typically dictated by their amino acid sequence within the parent protein and their composition [85]. Macroalgae-derived peptides include those proven to reduce hypertension sold as Ameal-S 120^®^, produced by Calpis Company, Tokyo, Japan, and Evolus ^®^ by Valio Ltd., Helsinki, Finland [86].

MAAs are considered important antioxidants in Rhodophyta and are able to absorb UV-B radiation, giving them high potential as bioactive ingredients in sun-protective creams [85]. Lectins from Rhodophyta and Chlorophyta have been shown to have anti-inflammatory results in studies, as well as acting as an antibacterial and antiviral and having pro-healing qualities and other antimicrobial properties [87].

Beyond high-molecular-weight biomolecules, marine macroalgae produce a diverse repertoire of low-molecular-weight secondary metabolites (typically <1500 Da) that play critical ecological roles in defence and signalling and increasingly represent promising leads for biomedical applications [9,22]. These include terpenoids, halogenated compounds, phenolic derivatives, and small bioactive lipophilic molecules [22].

Among these, terpenoids are one of the most structurally diverse and biologically active classes identified in macroalgae, particularly within Phaeophyceae [22]. Brown algae such as *Bifurcaria bifurcata* are well recognised for their production of linear and cyclic diterpenes, including compounds such as eleganolone, bifurcadiol, and related meroditerpenoids, which have demonstrated cytotoxic, antioxidant, and anti-proliferative activities across cancer cell models [35].

These compounds are biosynthetically derived from isoprenoid pathways and exhibit activity that is often linked to membrane interaction, redox modulation, and induction of apoptosis. Importantly, variability in their abundance is strongly influenced by environmental conditions, reinforcing the link between ecological stress and metabolite diversity [26].

In addition to diterpenes, macroalgae—particularly red algae (Rhodophyta), produce a wide range of halogenated monoterpenes and sesquiterpenes, especially within genera such as Laurencia and Plocamium [33]. These compounds frequently contain bromine or chlorine substituents and are associated with potent anti-inflammatory, antimicrobial, and anti-proliferative activities [88]. Mechanistically, their bioactivity has been linked to the disruption of cellular membranes, modulation of intracellular signalling pathways, and interference with enzymatic processes [88]. The degree and position of halogenation are known to significantly influence biological activity, suggesting clear structure–activity relationships that may be exploited for drug development [34].

Other macroalgal secondary metabolites include small phenolic derivatives and lipid-associated compounds, which contribute to antioxidant defence and immunomodulation [8]. Although less extensively characterised than polysaccharides or pigments, these low-molecular-weight compounds provide an important complementary layer of bioactivity, often acting synergistically within crude extracts [9,41].

#### 3.4.2. Peptides and Secondary Metabolites in Microalgae

Microalgae also produce bioactive peptides and secondary metabolites with antimicrobial, anticancer, and anti-inflammatory properties [89]. For example, peptides derived from *Chlorella vulgaris* protein hydrolysates have demonstrated antioxidant and antihypertensive activities [90]; while compounds isolated from dinoflagellates such as *Amphidinium* spp. include amphidinols, which exhibit potent antifungal and cytotoxic activities [91]. These molecules often arise from stress-induced metabolic pathways and may function as defence compounds or signalling molecules within marine ecosystems. Increasing interest in metabolomics and bioprospecting of marine microalgae continues to reveal new classes of secondary metabolites with high potential in medical applications.

Microalgae and associated microorganisms further expand the chemical diversity of low-molecular-weight metabolites, particularly through the production of polyketides, alkaloids, and toxin-derived bioactives [7,92]. These compounds are typically synthesised via polyketide synthase (PKS) or non-ribosomal peptide pathways and frequently exhibit high potency, albeit sometimes with associated toxicity that limits direct therapeutic use [93].

A key class within this category is microalgal polyketides, exemplified by compounds such as okadaic acid and amphidinolides, produced by dinoflagellates [50]. Okadaic acid is a well characterised inhibitor of serine/threonine protein phosphatases (PP1 and PP2A), leading to dysregulation of phosphorylation-dependent signalling pathways, induction of oxidative stress, and apoptosis [94]. While its toxicity precludes direct clinical application, it remains an important molecular tool in cancer and cell-signalling research. Amphidinolides, in contrast, represent a structurally diverse group of macrolide polyketides with potent cytotoxic activity, often acting through disruption of cytoskeletal organisation and actin dynamics [50,91].

Microalgae also produce bioactive alkaloids and neurotoxins, including domoic acid and saxitoxin-like compounds, which interact with ion channels or neurotransmitter receptors [92]. Domoic acid, for example, acts as a glutamate receptor agonist, inducing excitotoxicity through overstimulation of neuronal signalling pathways. Although primarily studied in the context of harmful algal blooms, these compounds provide valuable insight into receptor-targeted pharmacology and highlight the potential of marine metabolites as highly specific bioactive scaffolds [93].

Importantly, despite their toxicity, these microalgal metabolites demonstrate clear mechanistic precision, targeting defined molecular pathways such as phosphatase inhibition, ion channel modulation, or cytoskeletal disruption [92,94]. This specificity underpins their potential utility as lead structures for drug development, provided that issues related to toxicity, selectivity, and delivery can be addressed [93].

### 3.5. Common Biological Activities Across Classes

#### 3.5.1. Anti-Inflammatory and Antioxidant Effects

Marine macroalgae and microalgae represent a well-established source of anti-inflammatory and antioxidant agents spanning diverse functional classes, including carotenoids, phenolics (e.g., phlorotannins), sulphated polysaccharides, glycolipids, and sterols. Across in vitro and in vivo systems, these metabolites consistently converge on the suppression of key pro-inflammatory mediators, such as nitric oxide (NO), prostaglandin E_2_ (PGE_2_), TNF-α, IL-6, and IL-1β, and the downregulation of inducible enzymes including iNOS and COX-2. These effects are typically accompanied by restoration of redox homeostasis through coordinated inhibition of NF-κB and MAPK signalling pathways and activation of Nrf2-dependent antioxidant responses [95] (Table 1). Importantly, while this mechanistic convergence is consistent, biological activity is strongly influenced by physicochemical properties, including molecular weight, degree of sulphation or polymerisation, lipid unsaturation, and compound stability. These parameters govern cellular uptake, receptor interaction, and metabolic fate, thereby shaping overall bioactivity and translational potential.

Within this shared framework, carotenoid pigments are among the most extensively characterised contributors. Fucoxanthin (macroalgae) and astaxanthin (microalgae) demonstrate robust anti-inflammatory and antioxidant activity through inhibition of NF-κB signalling and enhancement of Nrf2-mediated cytoprotective pathways [95,96,97,98]. However, their translational potential is conditioned by metabolic transformation and physicochemical stability; for example, fucoxanthin is converted to fucoxanthinol during absorption [99], while its stability is influenced by environmental factors such as pH, temperature, and light [100]. In contrast, for astaxanthin, stereochemistry and esterification play a key role in determining stability and bioavailability, with esterified forms often showing improved stability under digestion-relevant conditions [101]. These considerations highlight the need to evaluate metabolite profiles and formulation strategies alongside intrinsic bioactivity (Table 1).

Phenolic compounds, particularly phlorotannins from brown macroalgae, further reinforce this paradigm by combining direct radical scavenging with pathway-level immunomodulation [102]. Their activity is influenced by the degree of polymerisation and extract composition, with lower-molecular-weight subfractions often exhibiting enhanced cellular activity. Across various studies the inhibition of NF-κB and MAPK signalling is consistently observed, resulting in reduced expression of iNOS, COX-2, and pro-inflammatory cytokines (Table 1) [103,104].

Sulphated polysaccharides, including fucoidan, laminarin, and ulvan, represent a structurally complex class in which bioactivity is highly dependent on molecular architecture. Variations in sulphation pattern and molecular weight distribution influence receptor interactions, biodistribution, and overall biological effect [105]. Fucoidan, for example, comprises heterogeneous sulphated polysaccharide populations whose anti-inflammatory efficacy, demonstrated across cell-based and in vivo models, is closely linked to these structural features [106,107], while low-molecular-weight ulvan fractions show improved efficacy and tolerability in vivo [104]. Laminarin further illustrates the role of biological context, acting as both an antioxidant and an immunomodulator depending on molecular characteristics and receptor engagement [108,109] (Table 1).

Lipid-derived compounds, including sterols, glycolipids, and polyunsaturated fatty acids, also contribute to anti-inflammatory and antioxidant effects through the modulation of NF-κB, MAPK, and Nrf2 pathways. Here, activity is frequently associated with the degree and positioning of unsaturation, which influences membrane interactions and downstream signalling [110]. Compounds such as fucosterol, glycolipid fractions (e.g., MGDGs), and microalgae-derived fatty acids (e.g., DGLA) demonstrate suppression of inflammatory mediators alongside modulation of lipid signalling pathways, reinforcing the link between lipid structure and bioactivity (Table 1) [111,112,113].

Across these classes, several studies report favourable selectivity and tolerability within active concentration ranges, particularly for polysaccharides and lipid-derived compounds. In vivo evidence further supports translational relevance, with fucoidan demonstrating efficacy in acute and systemic inflammation models [106,107], ulvan improving outcomes in colitis [114], and laminarin providing protection in UV-induced skin injury [109]. However, context-dependent effects, particularly for immunomodulatory polysaccharides such as laminarin, highlight the importance of defining the molecular characteristics and biological setting when interpreting outcomes [108].

Collectively, algal-derived bioactive compounds converge on the suppression of inflammatory signalling and restoration of redox balance, with their activity strongly influenced by structure–activity relationships and physicochemical properties [115,116]. Key translational challenges include standardisation, molecular characterisation, and bioavailability, particularly for carotenoids, as well as limited cross-study comparability, underscoring the need for harmonised reporting to support clinical and biotechnological translation [117,118,119].

**Table 1:** Representative anti-inflammatory and antioxidant effects of marine algae. The down arrow (↓) indicates a decreasing change, while the up arrow (↑) indicates an increasing change.

Functional Class	Compound/Extract	Representative Chemical	Source Species	Experimental Model	Assay/Effect Size or IC_50_ (Units)	Mechanism(s)	Key Ref(s)
Carotenoids (Pigments)	Fucoxanthin	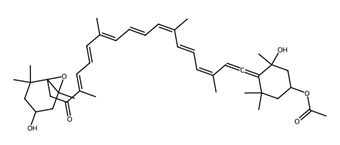	*Ishige okamurae*	LPS-stimulated RAW 264.7 macrophages	↓ NO and PGE_2_; ↓ iNOS/COX-2 proteins; ↓ IL-1β/TNF-α/IL-6 (dose-dependent)	↓ NF-κB activation (inhibition of IκBα degradation and p50/p65 translocation); ↓ MAPK phosphorylation (ERK/JNK/p38)	[96]
	Fucoxanthin	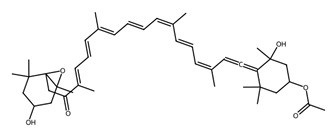	Brown algae fractionation (*Myagropsis myagroides* identified as source for active fraction)	LPS-stimulated RAW 264.7 macrophages	NO inhibition correlated with fucoxanthin abundance (r^2^ = 0.9511); ↓ iNOS/COX-2 proteins and gene expression. ↓ IL-1β/TNF-α/IL-6 (dose-dependent)	Anti-inflammatory profile dominated by iNOS downregulation	[97]
	Astaxanthin	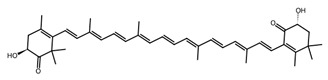	*Haematococcus pluvialis*	RAW 264.7 macrophages; WT vs. Nrf2-deficient BMDMs	↓ Il-6 and Il-1β mRNA; ↓ ROS; ↓ NF-κB p65 nuclear translocation	NF-κB inhibition; NRF2 nuclear translocation; NOX2 downregulation; altered macrophage polarisation. Nrf2-dependent and Nrf2-independent pathways	[98]
Glycolipids	MGDGs (1:1 mixture) and monoacylglycerol	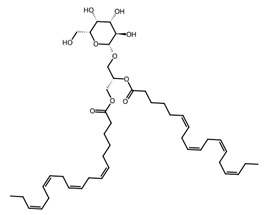	*Fucus spiralis*	LPS-stimulated RAW 264.7	NO inhibition IC_50_: 60.06 µg/mL (MGDG mixture) vs. 65.70 µg/mL (monoacylglycerol); unsaturation linked to potency	iNOS-linked NO suppression; SAR suggests higher unsaturation improves activity	[112]
Lipids (fatty acids)	DGLA (free acid/ethyl ester; microalga-derived preparation)	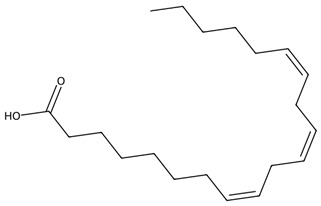	*Lobosphaera incisa* P127	LPS-stimulated RAW 264.7	100 µM: ↓ NO and IL-6; ↓ iNOS/IL6 transcription; non-toxic up to 250 µM	Prostanoid shift towards PGE1; ↓ NO/iNOS; ↓ ROS in LPS context	[113]
Phlorotannins/phenolics	Phlorotannin-rich extract and fractions	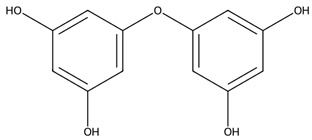 e.g., Fucophlorethol A	*Fucus vesiculosus*	Chemical antioxidant assays; LPS-stimulated RAW 264.7	NO• scavenging IC_50_ 75.2 ± 5.1 µg/mL (crude); O_2_•^−^ IC_50_ 98.7 ± 11.1 µg/mL; XO IC_50_ 2.8 ± 0.4 µg/mL; at 100 µg/mL, NO• reduced to ~14–17% vs. LPS	Lower-MW fractions more active; NF-κB blockade via inhibition of IκBα phosphorylation/degradation	[102]
	Phlorofucofuroeckol A	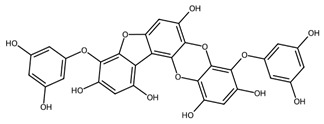	*Ecklonia stolonifera*	LPS-treated RAW 264.7	20 µM: ↓ iNOS/COX-2 mRNA; ↓ IL-1β/IL-6/TNF-α	↓ NF-κB and AP-1 promoter activity; ↓ Akt and p38 MAPK activation	[103]
Polysaccharides	Fucoidan fraction “SF6”	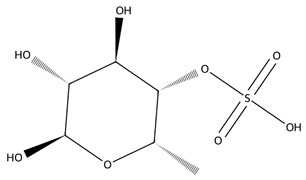 α-L-fucose-4-sulphate (fucoidan repeat)	*Saccharina japonica*	LPS-stimulated RAW 264.7	Viability >90% at 50–200 µg/mL; 200 µg/mL: NO inhibition ~dexamethasone comparator; ↓ iNOS/COX-2; ↓ TNF-α/IL-6/IL-1β	↓ NF-κB activation (IKK/IκB; p50/p65 nuclear translocation); ↓ MAPKs; ↓ JAK2–STAT1/3	[106]
	Fucoidan (structurally characterised; multi-MW fractions)	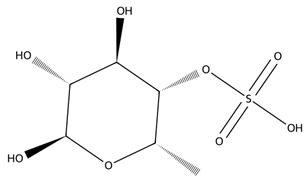 α-L-fucose-4-sulphate (fucoidan repeat)	*Cystoseira/Ericaria crinita*	Histamine paw oedema rat; LPS systemic inflammation rat	Paw oedema markedly reduced at 25–50 mg/kg; serum IL-1β and TNF-α reduced	In vivo anti-inflammatory effect with cytokine downregulation	[120]
	Low-molecular-weight (2.6 kDa) sulphated ulvan	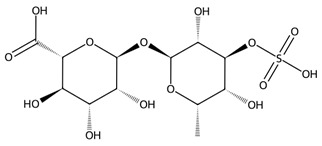 Ulvanobiouronic acid (ulvan repeat)	*Ulva* sp.	DSS-induced colitis mouse model	50 and 100 mg/kg: ↓ DAI; ↓ colon shortening; ↓ MDA; ↑ GPx and CAT; acute toxicity absent <1200 mg/kg	Antioxidant defence restoration; inflammation reduction; barrier/tight-junction support	[114,121]
	Laminarin	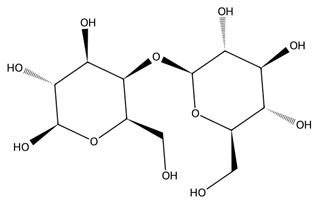 Laminaribiose (laminarin repeat)	*Laminaria digitata*	UVB-induced dorsal-skin damage in mice	3% Topical administration: ↓ superoxide-associated oxidative stress; ↑ SOD1/SOD2, GPx, catalase; improved histology	Antioxidant enzyme restoration linked to reduced oxidative injury and skin inflammation features	[109]
Sterols	Fucosterol	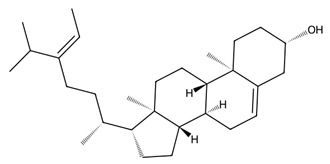	*Undaria pinnatifida*	LPS-induced RAW 264.7	↓ NO, TNF-α, IL-6 via transcriptional downregulation (concentrations not stated in abstract)	↓ NF-κB DNA binding/transcriptional activity; ↓ NF-κB phosphorylation/nuclear translocation; ↓ p38 pathway signalling (MKK3/6, MK2)	[122]
	Fucosterol	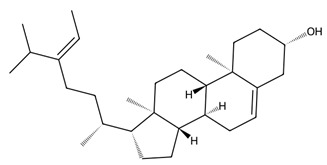	*Sargassum horneri*	TNF-α/IFN-γ-stimulated human dermal fibroblasts	Non-toxic up to 120 µM; 30/60/120 µM: ↓ ROS; ↑ viability vs. cytokine-stressed cells	↑ Nrf2 nuclear translocation; ↑ HO-1/NQO1; ↓ NF-κB/MAPK phosphorylation and p65 nuclear translocation	[123]

#### 3.5.2. Cytotoxic and Anti-Proliferative Activity

Marine macroalgae and microalgae also exhibit cytotoxic and anti-proliferative activity across multiple functional classes, including carotenoids, phenolics, sulphated polysaccharides, terpenes, and polyketides [124]. Despite chemical diversity, these effects consistently converge on apoptosis induction and cell-cycle disruption, often involving mitochondrial pathways, caspase activation, and modulation of MAPK and PI3K/AKT signalling [124,125]. As for anti-inflammatory activity, cytotoxic effects are strongly influenced by physicochemical properties, including molecular weight, sulphation pattern, lipophilicity, and compound composition, which determine potency, selectivity, and cellular uptake [93].

Within this framework, carotenoids are among the best characterised algal cytotoxic agents, demonstrating reproducible anti-proliferative effects across multiple cancer cell models both in vitro and in vivo [122,123,124]. These effects are typically associated with ROS generation, mitochondrial dysfunction, and pro- and anti-apoptotic proteins, highlighting a central role for intrinsic apoptotic pathways. Importantly, activity is influenced by the isomeric composition, co-extracted metabolites, and bioavailability constraints, meaning that purified compounds and complex extracts may not be biologically equivalent [126,127,128].

Phenolic compounds, particularly phlorotannins, reinforce this mechanistic pattern through combined redox modulation and signalling disruption. Evidence from in vitro and in vivo systems supports their ability to induce apoptosis via mitochondrial pathways and caspase activation [125], with activity influenced by structural features such as degree of polymerisation.

Beyond these classes, secondary metabolites such as alkaloids, terpenes, and polyketides expand the anti-proliferative landscape while maintaining mechanistic convergence. These compounds frequently act through apoptosis-associated pathways, including caspase activation, ROS generation, and cytoskeletal disruption [126,127]. In particular, polyketide metabolites exhibit high potency but remain translationally constrained due to toxicity [129,130].

Polysaccharides, including fucoidan, laminarin, and ulvan, generally act at higher concentrations but retain consistent anti-proliferative effects, often associated with apoptosis and cytostatic regulation [128,131]. Their activity is strongly dependent on structural features such as molecular weight and sulphation pattern, with emerging roles in combination therapies through modulation of ROS- and AKT-related signalling.

Extract-based studies further highlight the importance of experimental context. Differences between 2D and 3D models, alongside variability in cultivation conditions, significantly influence observed bioactivity [41,132,133]. These findings reinforce the need for physiologically relevant systems when assessing translational potential.

Overall, apoptosis remains the dominant anti-proliferative endpoint across algal classes, typically coupled with redox modulation and cell-cycle disruption. However, translational potential is shaped by variability in potency, selectivity, and physicochemical properties, alongside limitations related to the extract heterogeneity, bioavailability, and toxicity of highly potent metabolites (Table 2) [119,120,121,122,123,124,125,126,127,128,129,130,131,132,133,134]. Greater standardisation of compound characterisation and model systems will be critical for advancing these compounds towards clinical application [125].

#### 3.5.3. Pro-Regenerative and Protective Effects

Marine macroalgae and microalgae also exhibit consistent pro-regenerative and tissue-protective activity across key functional classes, particularly sulphated polysaccharides, carotenoids, and sterols [135,136]. These activities are mediated through integrated modulation of oxidative stress, inflammation, and regenerative signalling pathways. Key functional outcomes include enhanced wound healing, promotion of angiogenesis (VEGF/eNOS), and regulation of extracellular matrix remodelling, alongside reductions in oxidative stress and inflammatory mediators [137,138,139]. These effects extend across ischaemic and inflammatory models, where improvements in perfusion, endothelial function, and barrier integrity are consistently observed.

Carotenoids and related compounds further reinforce this paradigm through combined antioxidant and anti-inflammatory mechanisms, particularly in photodamage models, where they preserve collagen structure and restore endogenous antioxidant defences [140,141,142]. At a systemic level, compounds such as fucoidan mitigate organ injury by suppressing pro-inflammatory cytokines and apoptosis while activating Nrf2-dependent antioxidant pathways [143,144]. Sterols such as fucosterol similarly contribute to maintaining fibroblast function under inflammatory stress through coordinated modulation of redox and signalling pathways [119].

Collectively, these findings position algal bioactives as multifunctional modulators of tissue repair, acting at the interface of oxidative stress, inflammation, and regenerative signalling [136,137]. However, as with other activity domains, translational potential remains dependent on physicochemical properties, bioavailability, and standardisation, underscoring the need for well characterised compounds and clinically relevant models.

## 4. Translational Applications of Algae-Derived Bioactives

Section four explores the translational applications of algae-derived bioactives across nutrition, skincare, regenerative medicine and oncology. Examples of relevant clinical trials and their status are available in Appendix A, Table A1.

### 4.1. Nutraceuticals and Food Security

Beyond their role as sources of diverse bioactive metabolites, macroalgae and microalgae are increasingly recognised as valuable nutraceutical ingredients and functional food resources with relevance to chronic disease prevention, dietary quality, and food security [9,16]. Their potential is underpinned by a rich composition that includes long-chain polyunsaturated fatty acids, carotenoids, sulphated polysaccharides, phenolics, peptides, vitamins, minerals, fibre-like carbohydrates, and high-quality protein [145]. Accordingly, recent reviews position marine algae as multifunctional nutritional assets with clear translational relevance to preventive health [16,145].

From a nutraceutical perspective, the strongest evidence relates to cardiometabolic health. Meta-analyses show that edible algae, particularly *Spirulina*, can reduce blood pressure, while supplements from macroalgae improve body mass index, fat mass, total cholesterol, and low-density lipoprotein cholesterol, although glycaemic effects remain less consistent [140,141]. These findings support the use of marine algae as a preventive nutritional intervention rather than a therapeutic substitute, particularly in relation to hypertension, dyslipidaemia, and obesity-associated metabolic dysfunction [140,141].

The preventive effects of marine algae likely arise from the chemical complexity of their biomass. Macroalgae provide diverse polysaccharides, pigments, polyphenols, minerals, and lipids, while microalgae are important sources of EPA- and DHA-rich oils, protein concentrates, and carotenoid-rich ingredients [142]. This translational potential is already reflected in commercially relevant products, including omega-3-rich oils, whole-biomass powders, phycocyanin extracts, and functional food or feed ingredients from genera such as *Chlorella*, *Nannochloropsis*, *Schizochytrium*, and *Arthrospira* (*Spirulina*) [16,142,143,145]. Accordingly, marine algae now occupy an active innovation space spanning nutraceuticals, fortified foods, aquafeed, and dietary supplements [9,16,142,143,145].

A further preventive mechanism involves the gut microbiome. Many macroalgal polysaccharides are resistant to digestion by host enzymes yet remain accessible to microbial fermentation, thereby influencing the generation of metabolites relevant to inflammation, oxidative balance, satiety, and lipid and glucose homeostasis [144]. On this basis, macroalgal-derived polysaccharides are increasingly regarded as promising prebiotic substrates, particularly in the context of metabolic syndrome and associated chronic disorders [144].

Microalgae are also highly relevant to food security, particularly at the systems level. Unlike many conventional food and feed resources, microalgae can be cultivated on non-arable land, may use saline or non-potable water, and can be integrated into circular bio-economy models involving carbon capture, wastewater valorisation, and nutrient recovery [146]. They are therefore attracting increasing interest as alternative protein sources and as sustainable ingredients for animal feed and aquaculture [147]. This trajectory is also reflected in the development of the European algae sector, where 447 algae and *Spirulina* production units across 23 countries have been identified, with biomass directed primarily towards food, food-related applications, supplements, and nutraceuticals [147].

However, this potential must be considered alongside important translational constraints, including compositional variability, production costs, scalability, consumer acceptance, and safety assurance [9,16,145]. Macroalgae may accumulate heavy metals, iodine, and other contaminants, while microalgae-based products still face challenges in cultivation, downstream processing, and market uptake [9,16]. Progress will therefore require standardised cultivation and processing, species-specific compositional profiling, contaminant monitoring, and stronger human evidence based on well-defined preparations and doses [148]. The commercial implications of this are discussed further in Section 5.1.2.

Overall, marine algae represent a versatile platform for preventive nutrition and more resilient food systems. Their value lies not only in their bioactive richness, but also in their capacity to support more sustainable and health-oriented models of production and consumption [16,145]. With robust evidence, safety assurance, and technological standardisation, they could make a meaningful contribution to chronic disease prevention, dietary diversification, and food security.

### 4.2. Translational Potential of Marine-Derived Bioactives in Cosmetics

#### 4.2.1. Molecular Drivers of Skin Ageing and Cosmetic Intervention Points

Human skin, being in contact with the external environment, is continuously exposed to numerous harmful agents, driving its ageing and deteriorating its appearance. Among these agents, solar UV exposure is the major generator of reactive oxygen species (ROS) mediating oxidative stress within the skin [149,150]. Consequently, solar exposure triggers hyperpigmentation (excessive production of melanin), dryness, and the degradation of extracellular matrix (ECM) proteins, forming wrinkles [149,151].

The cosmetics industry has an established demand for developing innovative anti-ageing products, most recently focused on achieving the following outcomes: (i) photoprotection against solar exposure through antioxidant activity; (ii) skin whitening by targeting tyrosinase (TYR), a rate-limiting enzyme in melanogenesis; and (iii) skin firmness by targeting matrix metalloproteinases (MMPs) and UV-inducible enzymes degrading ECM proteins [149,150].

Marine ecosystems are a particularly attractive resource for cosmetics as they contain a vast, underexploited library of structurally diverse molecules produced by marine organisms to adapt to prominent environmental stressors, namely UV exposure [149,150]. As discussed throughout Section 3, marine-sourced actives are commonly organised into several functional classes. In cosmetics MAAs, polysaccharides, carotenoids, polyphenols, fatty acids, peptides/proteins, alkaloids, and terpenoids, each map onto specific, translationally relevant skin endpoints [149].

#### 4.2.2. Photoprotection: UV Absorption and ROS Control

As photoaging is strongly tied to UV-induced ROS and subsequent tissue damage, one translational opportunity is to deploy marine compounds either as UV filters or as bioactives that attenuate UV-induced cellular damage through a combination of UV-absorbing capacity and radical scavenging properties [151]. Anti-photoaging activity is tightly linked to a combination of UV-absorbing capacity and radical scavenging properties. Macroalgae, found from intertidal shores down to ~150 m depth, experience substantial UV exposure and therefore synthesise photoprotective molecules [150]. MAAs are photostable molecules with additional antioxidant roles that absorb UVA/UVB and dissipate energy as harmless heat without photochemical reactions [150,152,153]. In vivo, MAA-containing emulsions (mycosporin-2-glycin, shinorine, porphyra-334) applied to mouse ears upregulated antioxidant enzyme activity and showed anti-photooxidative abilities upon irradiation [154]. In vitro, palythine, an MAA extracted from the red algae *Chondrus yendoi*, is reported to afford broad protection in keratinocytes exposed to solar-simulating and UVA radiation, including protection against DNA photolesions (cyclobutane pyrimidine dimers and 8-oxo-7,8-dihydroguanine) [152]. Collectively, these findings support the framing of MAAs as potential natural, biocompatible alternatives to approved UV filters, and as an eco-friendly route to photoprotection.

Macroalgal polysaccharides (fucoidan, laminarin, carrageenan) are similarly positioned as anti-photoaging ingredients, with effects that are mediated by intracellular ROS scavenging in UV-irradiated cells and in vivo models, alongside moisturising benefits that help boost skin appearance [150]. For example, fucoidan photoprotection has been studied in UVB-irradiated HaCaT keratinocytes, human foreskin fibroblasts (HS 68), zebrafish, and in other in vivo models, supporting its potential relevance to the skincare and cosmetic industries [151]. Carrageenans tested in UVB-irradiated HaCaT cells provided significant protection against UVB-induced apoptosis and scavenged free radicals [155]. Laminarin treatment in mice models increased dermal thickness, decreased MMP-1 expression, protected dorsal skin from UVB-induced photodamage, and increased collagen fibres in UVB-treated skin [156]. Laminarin also attenuated IL-6 and ROS levels in UVA-irradiated dermal fibroblasts and keratinocyte models [150]. Such polysaccharide examples map onto a coherent translational axis: controlling ROS reduces inflammation and MMPs activity, which in turn preserves ECM structure, all aligning with anti-photoaging claims.

Additional photoprotective opportunities arise from polyphenols, particularly phlorotannins and carotenoids which show photoprotective properties, especially against UVB. Phlorotannins are potent radical scavengers, where hydroxyl groups donate electrons to reactive species, limiting ROS-mediated macromolecule damage and inhibiting signalling pathways such as NF-κB and MAPK [150,157]. In fact, phenolic compounds from *Turbinaria ornata* showed antioxidant activity and very high tyrosinase-inhibiting activity, while an ethyl acetate fraction from *Padina boergesenii* demonstrated antioxidant and UV-shielding effects in keratinocytes upon UV-induced damage [149,158]. Carotenoid-rich extracts (e.g., from *Padina australis*) showed antioxidant activity and protected human keratinocytes from UV-induced damage [149]. However, a notable key limitation is that cutaneous absorption of marine carotenoids remains to be determined, a crucial step before their implementation in sunscreen formulations.

#### 4.2.3. Pigmentation Control: TYR Inhibition as a Dominant Cellular Target

Within a cosmetics context, equalising pigmentation is linked to the targeting of tyrosinase, described as the dominant enzyme in melanogenesis. Several marine-derived compounds support this aim [149,150]. Red-algae polysaccharides (including 3,6-anhydrogalactose-containing fractions) from *Porphyra haitanensis*, *Gracilaria chouae*, and *Gracilaria blodgettii* are noted to have skin-whitening and tyrosinase-inhibiting activities [149]. Studies have found that phenolics from the Phaeophyceae species *Turbinaria ornata* were associated with very high tyrosinase inhibition, supporting dual anti-ageing/whitening properties [158]. Concerning marine by-products, astaxanthin derived from shrimp shells (*Litopenaeus vannamei*) inhibited TYR in a dose-dependent manner (3–50 g/mL), and a polypeptide hydrolysate from tilapia by-products suppressed TYR activity (5 g/mL) and reduced melanin production in mouse melanoma cells (B16-F10) [159,160]. A further example within phlorotannins includes the in vitro and in vivo whitening effects of 4-hydroxyphenethyl alcohol (4-HPEA) isolated from *Hizikia fusiformis*, where topical application promoted depigmentation of UVB-induced hyperpigmented spots in brown guinea pig skin [157].

Together, these studies support a translational narrative in which marine-derived compounds serve as anti-pigmentary candidates that converge on a well-defined molecular target (TYR) while remaining compatible with broader anti-photoaging strategies.

#### 4.2.4. Repair and Regeneration: Wound Healing and ECM-Centric Approaches

Beyond photoprotection, there exist marine-derived opportunities for skin repair through wound healing and ECM restoration. Wound healing is described as a physiological process occurring in three overlapping phases: inflammation, proliferation (encompassing angiogenesis, collagen deposition, formation of granulation tissue, and epithelialization), and tissue remodelling. Prolonged inflammatory cytokine secretion (e.g., TNF-α, IL-1) can extend the inflammatory phase and promote chronic wounds or hypertrophic scarring, while inflammatory mediators and free radicals increase tissue damage [161].

A key example is the sulphated polysaccharide low-molecular-weight fucoidan (LMF), which has been validated in a full-thickness dermal excision rat model. LMF showed beneficial effects comparable to or better than a commercial reference product and was linked to reduced neutrophil adhesion/leukocyte recruitment and inhibition of pro-inflammatory cytokine release. LMF treatment increased immunoreactive cells expressing TGF-β and VEGFR2 (associated with VEGF responses), inhibited TNF-α/IL-6/IL-1β expression, reduced iNOS and COX-2 expression, and decreased oxidative stress [161]. Altogether, LMF has potential as a topical agent to promote wound healing in humans. Complementing this, polysaccharides from *Gracilaria lemaneiformis* (GLP-2) enhanced keratinocyte proliferation and migration, consistent with a wound-healing product rationale [149]. Additionally, laminarin-based creams are linked to accelerated collagen deposition and re-epithelialization in rats and protected skin cells from oxidative stress [162].

#### 4.2.5. Translational Considerations: Delivery Routes, Advanced Testing Models, and Sustainable Sourcing

With the growing demand to incorporate natural compounds, the translational success of marine-derived compounds depends not only on their bioactivity but also on their formulation, delivery, and safety. For example, despite high antioxidant activity, oral fucoxanthin did not result in efficient cutaneous concentrations in mice, motivating topical delivery. In a reconstructed human skin (RHS) model, built from a fibroblast-containing a type I collagen dermal compartment topped with keratinocytes and raised to an air–liquid interface, topical fucoxanthin at 0.5% (within the 0.01–1% range used for antioxidants in cosmetic formulations) was delivered in alkyl benzoate [163]. In this model, fucoxanthin ameliorated the detrimental effects of ethanol on tissue viability and inflammatory response, illustrating how advanced skin equivalents can support more physiological assessments aligned with cosmetic safety and efficacy evaluation [163].

### 4.3. Regenerative Medicine and Tissue Repair

Regenerative medicine encompasses a broad spectrum of strategies aimed at restoring tissue structure and function through the integration of biomaterials, bioactive compounds, and cell-based therapies. This therapeutic approach to treat degenerative diseases aims to repair damaged tissues by acting on progenitor or stem cells to restore physiological functions. Research in this field typically investigates the mechanisms underlying the differentiation of progenitor cells into mature entities. While applications span multiple organ systems, the musculoskeletal system represents a particularly relevant and challenging target due to the limited intrinsic regenerative capacity of cartilage, bone, and connective tissues, as well as the high prevalence of degenerative conditions such as osteoarthritis. In this context, the development of functional biomaterial platforms that can support cell localisation, modulate the microenvironment, and deliver bioactive cues is critical for therapeutic success. Marine algae-derived polysaccharides and bioactive metabolites are increasingly recognised as promising candidates within this landscape, with physicochemical properties and immunomodulatory and pro-regenerative activities that are directly relevant to musculoskeletal repair.

#### 4.3.1. Regenerative Medicine for Musculoskeletal Diseases

Disorders of the musculoskeletal system are leading contributors to disability worldwide, commonly affecting between one in three and one in five people from adolescence to old age. According to the World Health Organization, the most common musculoskeletal conditions are osteoarthritis, back and neck pain, fractures associated with osteoporosis and injuries, and autoimmune inflammatory diseases like rheumatoid arthritis. These conditions are typically associated with persistent pain, limited mobility and dexterity, and a reduction in people’s ability to work and participate in social roles, with a direct negative effect on mental wellbeing and communities’ health status [164].

Experiments investigating progenitor cells have addressed their use to repair bone, cartilage and other tissues compromised by chronic degeneration. The production of new tissue in vitro and in vivo is influenced by several factors such as the cell type involved, microenvironment and local stimulation by growth factors or other molecules. Gold-standard progenitor cells that can generate bone and cartilage are Mesenchymal Stem Cells (MSCs), undifferentiated cells with a fibroblastic shape that can differentiate to osteogenic, chondrogenic and adipose lineages [165]. The microenvironment stimulating their differentiation is extensively studied by tissue engineers, experimenting with various biomaterials such as collagen or marine-derived calcium phosphate matrices as scaffold structures to home cells that can facilitate their differentiation. Local stimulation to drive cell fate is also extensively studied, mostly using peptide growth factors such as Transforming Growth Factors (TGFβs) and Bone Morphogenic Proteins (BMPs) or compounds widely implemented for in vitro studies such as dexamethasone. Additional effort investigates the potential of new bioactive compounds to improve the stimulatory effect.

Since the early demonstration that MSCs were able to form multiple skeletal tissues in vivo [166], these cells gained interest for therapeutic use in regenerative medicine to repair damaged articular cartilage [167]. The initial concept was a “tissue-specific cell replacement therapy” [168], wherein MSCs delivered to damaged cartilage would differentiate into chondrocytes, while MSCs delivered to damaged bone would differentiate into osteogenic progenitors to specifically regenerate the required tissue under local stimulating factors. This strategy was approached in different ways: using autologous or allogeneic MSCs, in vitro expanded or stimulated cells for differentiation, and often implementing biomaterials or scaffolds such as collagen to improve cell retention in vivo after implantation. The initial idea of cell replacement therapy was demonstrated to be too simplistic over time and reliable tissue regeneration after cell injection was hindered by the lack of a clear understanding of the mechanisms influencing regeneration in situ. A significant step forward towards understanding the mechanism underlying in vivo tissue regeneration after MSC injection was made by studying an osteoarthritic joint model.

Osteoarthritis (OA) is the most common chronic condition of the joint affecting millions of people around the world and characterised by deterioration of cartilage and the development of undesired calcified structures causing severe pain. In osteoarthritis, the differentiation of endogenous stem cells to generate cartilage is too poor to maintain articular homeostasis, while other synovial stem cells differentiate along the osteogenic fate to generate abnormal bone formations and worsen the disease. A local inflammation state is also involved in the activation of pathways to influence osteogenic progenitors, generating a hostile environment for the correct physiological functioning of the joint. Pioneering work from Murphy et al. [169] showed amelioration of the osteoarthritic joint environment after intra-articular injection of autologous MSCs: typical signs of OA progression such as cartilage erosion, osteophyte formation, and subchondral sclerosis were significantly less evident in the cell-treated knees, but the effect was not obtained by differentiation of the injected MSCs.

#### 4.3.2. Regenerative Approaches to Other Degenerative Diseases

Studies in regenerative medicine are not only beneficial for articular cartilage regeneration or bone repair. The knowledge acquired on progenitor cell behaviour and their effect on the surrounding environment highlighted their role in several other degenerative pathologies. For example, development of the most common vascular disease, atherosclerosis, is strongly influenced by undesired behaviour of circulating progenitor/stem cells. Initially considered a passive degenerative process, vascular calcification developing in atherosclerosis is now considered an active and highly regulated disease involving progenitor cells. The aetiology of this condition is thought to begin with lipid accumulation or damage of the blood vessel which triggers the creation of the plaque. At this site circulating progenitor cells or pericytes in blood vessel walls proliferate, while local macrophages and monocytes secrete pro-inflammatory cytokines. The inflamed microenvironment with proliferating stem cells stimulates their abnormal differentiation and the production of extracellular matrix components such as collagen which contributes to plaque growth.

#### 4.3.3. Marine-Derived Compounds in Regenerative Medicine

Preclinical studies across skin, cartilage, and bone models consistently demonstrate multifunctional benefits, including enhanced wound closure, angiogenesis, and controlled delivery of bioactive factors, although these effects are often observed in composite systems and small-scale studies. Fucoidan-based materials, for example, have been shown to accelerate wound healing through angiogenic signalling pathways [170], while ulvan and carrageenan systems demonstrate improved repair when combined with antimicrobial or drug-delivery components [171,172]. Agarose-based composites similarly support bio-integration and vascularisation in vivo [173,174,175], underscoring the importance of hybrid material design in achieving functional regenerative outcomes. Importantly, within the musculoskeletal context, these biomaterial-driven strategies align with established regenerative medicine frameworks in which MSCs act as multipotent cells capable of differentiating into cartilage, bone, and other connective tissues central to skeletal repair and joint homeostasis [176,177]. In this setting, marine algae-derived biomaterials offer a strategic advantage by enabling both structural support and biochemical modulation of the MSC niche, including enhanced cell retention within cartilage defects, spatial organisation within bone matrices, and localised presentation of growth factors relevant to osteogenic and chondrogenic differentiation.

Emerging evidence suggests that sulphated polysaccharides such as fucoidan can influence key musculoskeletal regenerative pathways, including angiogenesis and osteogenesis, through interactions with VEGF and related signalling cascades [176,178,179], while their immunomodulatory properties may help mitigate the chronic inflammatory microenvironments characteristic of conditions such as osteoarthritis, which limit MSCs’ therapeutic efficacy [178,180]. This dual functionality, combining scaffold engineering with bioactive modulation, positions marine algae as a valuable component of next-generation musculoskeletal regenerative systems integrating biomaterials and cell-based therapies. Nevertheless, clinical translation remains uneven.

While alginate dressings are widely adopted, strong evidence of superiority over alternative treatments is limited in some indications [181,182], and more advanced applications such as injectable hydrogels highlight the need for long-term safety and efficacy data [183,184]. Across musculoskeletal applications, translational success is closely linked to purity, processing, and manufacturing considerations, including endotoxin control and sterilisation compatibility, which can significantly influence both material properties and biological responses [170,173,182,185,186,187,188].

Looking forward, the integration of well characterised marine algae-derived biomaterials with MSC-based and bioengineered musculoskeletal repair strategies is likely to be central to overcoming current translational bottlenecks, particularly through the development of standardised, scalable, and regulatory-compliant platforms capable of supporting clinically effective cartilage and bone regeneration.

### 4.4. Anticancer Therapeutics

Building on the conserved biological activities described in Section 3.3, bioactive compounds derived specifically from marine microalgae and macroalgae are increasingly recognised as a promising and underexploited source of anticancer agents. Their structural motifs, rare in natural terrestrial products (e.g., extensive sulphation, halogenated phenolics, polyunsaturated side chains), underpin potent activities against multiple hallmarks of cancer, ranging from cell-cycle dysregulation to tumour immune evasion [2,3,92]. Importantly, these metabolites often act through multi-target mechanisms rather than single molecular pathways, positioning them as particularly attractive candidates for addressing tumour heterogeneity and therapeutic resistance in hard-to-treat malignancies [64,92].

#### 4.4.1. Cell-Cycle Arrest and Apoptosis

Algal metabolites arrest the cancer cell cycle and initiate apoptosis through a spectrum of complementary mechanisms that map neatly onto their major chemical classes.

Carotenoids from brown algae are among the most potent agents in this context. Within cells, fucoxanthin is swiftly de-acetylated to fucoxanthinol, and together these two carotenoids downregulate cyclin D2/CDK4, inhibit PI3K/Akt and NF-κB, and activate caspases-9/3, culminating in G_1_ arrest and apoptosis across leukaemia, breast and cervical models [189,190,191]. The green-algal carotenoid siphonaxanthin penetrates cells even more efficiently; its additional hydroxyl group accelerates ROS-mediated mitochondrial damage and synergises with TRAIL to amplify apoptotic signalling [192].

Among sulphated polysaccharides, fucoidan exemplifies multi-target potency. By suppressing HIF-1α/VEGF it blocks EMT-driven angiogenesis, while simultaneous inhibition of the CXCR4 axis and Bcl-2 family proteins promotes intrinsic apoptosis in hepatocellular and leukaemia cells [193,194]. Laminarin, a β-glucan from *Laminaria digitata*, shifts the Bax/Bcl-2 ratio, releases cytochrome c, and attenuates ErbB2/3-PI3K signalling, leading to sub-G_1_ accumulation in colon cancer cells [195]. Their biological activity is strongly influenced by structural features such as molecular weight and sulphation patterns, which govern receptor interactions and downstream signalling responses. Structurally distinct ulvan (green algae) and κ-carrageenan (red algae) likewise enforce caspase-dependent death and G_2_/M arrest [196,197].

Among phenolics, polymeric phlorotannins unique to brown algae, dieckol, phloroglucinol, and eckstolonol, act as CDK1/2 inhibitors. They impose either G_2_/M or G_0_/G_1_ blockade and elicit ROS-driven apoptosis in breast, pancreatic and leukaemia models [102,198]. Their sizeable phenolic scaffolds also chelate transition metals, accentuating oxidative stress within tumour cells.

Phycobiliproteins and specialised pigments extend the arsenal of bioactive compounds isolated from marine algae with potential anticancer activity. R-phycoerythrin from red algae activates Fas and a cascade of caspases (-2, -3, -8, -9, -10), whereas C-phycocyanin from *Spirulina platensis* heightens Fas and ICAM expression on tumour membranes, fostering immune recognition while sparing normal cells [190]. The cyanobacterial pigment scytonemin inhibits polo-like kinase 1, locking multiple myeloma cells in G_2_/M [199].

Finally, cyclic and depsipeptidic peptides offer high-affinity microtubule or membrane targets. Kahalalide F disrupts membranes, arrests cells in G_0_/G_1_ and induces caspase-independent death, effects magnified in HER2/3-overexpressing tumour cells due to downregulation of Akt signalling [200]. Cryptophycin analogues from cyanobacteria bind tubulin with picomolar affinity, producing catastrophic spindle failure and apoptotic collapse [201].

Taken together, these diverse compounds converge on a unified outcome: decisive interruption of cell-cycle progression followed by programmed cell death. Their multi-target profiles, structural novelty and selective cytotoxicity provide a robust foundation for overcoming chemoresistance in aggressive, heterogenous tumours.

#### 4.4.2. DNA Damage and Repair Modulation

While most algal metabolites exert cytotoxicity through apoptosis and cell-cycle arrest, some can influence DNA integrity and repair processes, primarily through indirect mechanisms linked to oxidative stress and mitochondrial dysfunction. R-phycoerythrin causes DNA breakage alongside caspase activation, illustrating that algal phycobiliproteins can damage DNA [202]. Yessotoxins induce DNA fragmentation and perturb mitochondrial membrane potential [203]. This pro-oxidant shift, although context-dependent, can sensitise cancer cells to apoptosis and enhance susceptibility to conventional chemotherapeutic agents.

Polysaccharides such as fucoidan have also been implicated in modulating DNA repair pathways and chromatin organisation, although these effects remain less well characterised and appear to depend on structural heterogeneity and cellular context [204]. Rather than acting as direct genotoxic agents, algal-derived compounds are therefore better understood as modulators of cellular stress responses that indirectly compromise genomic stability in cancer cells. This capacity to enhance DNA damage or interfere with repair mechanisms supports their emerging role as potential chemosensitisers, particularly in combination with DNA-damaging agents, and highlights a key opportunity for integrating algal bioactives into combination therapy strategies [190].

#### 4.4.3. Immune and Tumour Microenvironment Modulation

Beyond direct cytotoxic effects, a defining feature of algal-derived bioactives is their ability to modulate the tumour microenvironment and immune responses, an area of increasing importance in contemporary oncology. Sulphated polysaccharides such as fucoidan and laminarin are known to interact with pattern recognition receptors and influence immune cell activation, including that of macrophages, dendritic cells, and natural killer cells [205,206]. These interactions can promote anti-tumour immunity through enhanced cytokine production, improved antigen presentation, and modulation of inflammatory signalling pathways.

In parallel, algal-derived compounds can regulate tumour-associated inflammation, a key driver of cancer progression. By suppressing pro-inflammatory mediators such as TNF-α, IL-6, and COX-2, mechanisms already described in Section 3.5, these bioactives may disrupt tumour-promoting signalling networks within the microenvironment.

These immunomodulatory and anti-inflammatory properties position algal-derived compounds as potential adjuncts to immunotherapy, with emerging evidence suggesting their ability to enhance immune surveillance and improve therapeutic responses. Importantly, this aligns with the broader concept of targeting both cancer cells and their surrounding microenvironment, rather than focusing solely on tumour-intrinsic pathways.

#### 4.4.4. Relevance to Unmet Clinical Need and Hard-to-Treat Cancers

Despite being at early developmental stages, several algae-derived compounds have progressed to clinical evaluation and show promise for cancers resistant to conventional therapies.

Fucoidan represents one of the most clinically advanced algae-derived polysaccharides, with multiple trials exploring its role primarily as a supportive therapeutic in oncology. A Phase II randomised, double-blind study in patients with stage III/IV head and neck squamous cell carcinoma, NCT04597476, is evaluating fucoidan administered alongside chemoradiotherapy [207]. This study, sponsored by Hi-Q Marine Biotech, enrolled 119 patients and is designed to assess both clinical outcomes and tolerability, providing one of the most robust indications that fucoidan is progressing beyond preclinical investigation into controlled clinical settings.

More recent trials further expand fucoidan’s application into supportive care and survivorship. A Phase II pilot study in cancer survivors, NCT06295588, led by the University of Rochester, is investigating an eight-week fucoidan supplementation regimen to reduce fatigue and systemic inflammation [208]. Similarly, a Phase II trial initiated by the Mayo Clinic, NCT06855524, is assessing whether fucoidan can mitigate chemotherapy-related fatigue in patients receiving platinum-based regimens for gastrointestinal and gynaecological cancers [209]. Together, these studies position fucoidan within an emerging paradigm of adjunctive therapies aimed at improving quality of life and immune recovery rather than acting solely as direct cytotoxic agents.

Earlier investigations have also explored fucoidan in the context of tumour-directed therapy and treatment-associated outcomes. In locally advanced rectal cancer, a double-blind, randomised placebo-controlled study, NCT04342949, evaluated fucoidan as an adjunct to neoadjuvant chemoradiotherapy, focusing primarily on quality-of-life endpoints rather than direct tumour response [210]. While supportive of clinical feasibility, this distinction highlights that much of the current clinical evidence for fucoidan remains centred on symptom management and treatment tolerance.

In hepatocellular carcinoma (HCC), a randomised, double-blind trial of oligo-fucoidan, NCT04066660, was designed to evaluate its role as supportive care in advanced disease [211]. However, this study was terminated early due to insufficient recruitment following the COVID-19 pandemic, underscoring the challenges associated with advancing marine-derived compounds through late-stage clinical evaluation.

Notably, attempts to extend fucoidan into other indications such as non-small-cell lung cancer have not yet translated into active clinical programmes. A pilot randomised study combining fucoidan with platinum-based chemotherapy, NCT03130829, was withdrawn prior to patient enrolment due to feasibility constraints [212], indicating that, despite strong preclinical rationale, clinical development remains selective and context-dependent.

Collectively, these trials demonstrate that fucoidan has achieved meaningful clinical traction, particularly in supportive oncology settings. While its direct anticancer efficacy remains to be established in large-scale interventional trials, its immunomodulatory, anti-inflammatory, and potential chemosensitising properties align closely with unmet clinical needs in advanced and treatment-resistant cancers. Importantly, the current clinical landscape suggests that fucoidan may be best positioned as part of combination or supportive strategies, rather than as a standalone cytotoxic agent, reinforcing the need for integrated trial designs and biomarker-driven patient stratification.

Beyond fucoidan, only a limited number of algae-derived compounds have progressed to clinical evaluation, and most remain at early or discontinued stages of development. Kahalalide F, a marine-derived cyclic depsipeptide originally isolated from the green alga *Bryopsis* spp., represents one of the earliest examples to enter clinical trials. Phase I and II studies demonstrated cytotoxic activity across several tumour types, including androgen-refractory prostate cancer, melanoma, and non-small-cell lung cancer, primarily through induction of G_0_/G_1_ cell-cycle arrest and caspase-independent cell death [213,214]. However, clinical development was hindered by dose-limiting toxicities, and no active oncology trials are currently ongoing. Similarly, Irvalec (PM02734), a synthetic marine-derived cyclodepsipeptide structurally related to Kahalalide F, has been evaluated in a Phase I trial in patients with advanced solid tumours (NCT00884845); however, its clinical development has since been discontinued despite initial signs of therapeutic potential [215]. These outcomes highlight both the potency and translational limitations of marine-derived cytotoxic peptides.

In contrast, phycocyanin, a phycobiliprotein derived primarily from cyanobacteria such as *Arthrospira platensis*, is being explored within a supportive and nutraceutical framework rather than as a direct anticancer agent. A Phase II clinical study (NCT05025826) is investigating its neuroprotective effects in patients with metastatic cancer receiving chemotherapy, specifically targeting chemotherapy-induced peripheral neuropathy [216]. This reflects its established antioxidant and anti-inflammatory properties and underscores a broader translational pathway for algal compounds, where clinical value may lie in reducing treatment-related toxicity and improving patient quality of life.

Collectively, the current clinical landscape indicates that marine-derived algal compounds are beginning to transition into human studies, albeit predominantly within supportive or early-phase contexts; a table of clinical trials and their status is available in Appendix A. While direct tumour-targeting efficacy remains limited, their immunomodulatory, anti-inflammatory, and protective properties align closely with unmet clinical needs in advanced and treatment-resistant cancers. These findings support a strategic repositioning of algal bioactives within combination therapies and supportive-care paradigms, alongside continued optimisation to enable progression towards fully therapeutic applications.

## 5. Challenges and Future Perspectives

Marine biodiscovery continues to develop as a central pillar of modern biotechnology, yet its successful translation into regulated markets is shaped by interconnected scientific, technical, and policy challenges. Although the marine environment offers an exceptional reservoir of structurally diverse bioactives with therapeutic, cosmetic, nutritional, and regenerative potential, the pathway from ecological discovery to commercial application is often fraught. To better understand these challenges, we convened a small focus group of seven Welsh and Irish SMEs specialising in marine natural products, representing the full innovation pipeline from algae production to medical product development and spanning a range of commercial maturity from pre-revenue start-ups to established companies with over 25 years of experience. Qualitative insights were collected through one-to-one interviews and a semi-structured group discussion.

These translational challenges must be considered within the context of a rapidly expanding global market for marine-derived products, which is driving both innovation and commercial pressure. The global market for algae and marine bioactives reflects steady and diversified growth across multiple high-value sectors. Recent estimates place the global algae products market at approximately USD 5.85–6.06 billion in 2025 (≈EUR 5.2–5.4 billion), expanding at a compound annual growth rate (CAGR) of ~5.8–6.7% [217,218], while the broader marine bioactives market was valued at around USD 2.4 billion in 2023 (≈EUR 2.1 billion) and is projected to grow at >5.6% CAGR through to 2032 [219]. Within this landscape, particularly strong demand is observed in high-value segments, including algae-derived omega-3 ingredients (USD 1.19 billion in 2024; 7.2% CAGR) [220], algae-based skincare products (USD 195 million in 2023; 7.2% CAGR) [221], and marine-derived pharmaceuticals, which represent the largest opportunity at approximately USD 8.4 billion in 2025 with a projected 5.7% CAGR [222]. Regulatory developments are also supporting market expansion and competitiveness, with the addition of over 20 algae species to the EU Novel Food Catalogue in 2024 [223], although market entry in Great Britain remains subject to Food Standards Agency authorisation under assimilated Regulation (EU) 2015/2283 [224].

**Figure 2:**
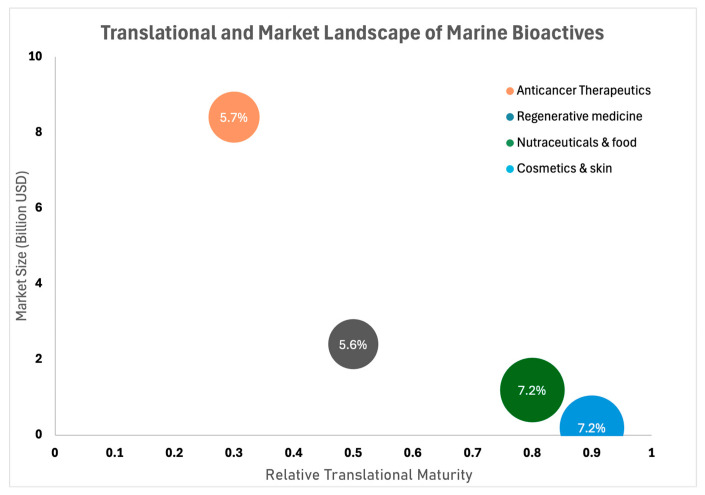
Translational and market landscape of marine-derived bioactives across key application domains. Bubble width reflects relative compound annual growth rates (CAGR), *x*-axis represents relative translational maturity (from early (0) to established (1)). Regenerative medicine is approximated using the broader marine bioactives market due to limited sector-specific estimates.

Collectively, these trends highlight a robust and scalable market trajectory, in which near-term opportunities in nutraceuticals and cosmetics provide commercially viable entry points capable of funding and de-risking longer-term development of marine-derived therapeutics. The scale, growth, and translational positioning of these application domains are summarised in Figure 2. However, realising this commercial potential depends on overcoming a series of interconnected translational challenges spanning compound variability, manufacturing, regulatory frameworks, and financing, with variability in marine-derived compounds emerging as a foundational constraint.

### 5.1. Variability and Standardisation of Marine-Derived Compounds

#### 5.1.1. Variability and Standardisation

Whilst diversity is exciting for bioprospecting and biodiscovery, the intrinsic variability of the marine environment, and therefore marine-derived compounds (as discussed in Section 2 and Section 3), presents a persistent translational challenge in health applications where consistency is key [225]. This is particularly relevant to macroalgae which demonstrate extensive variation in yield and composition with changing seasons and geographic distribution [225] and are typically wild-harvested or cultivated in situ, thus limiting the control of the diversity drivers. Batch-to-batch variability is demonstrated as fluctuating concentrations of key actives [226] and trace elements (vitamins and minerals) [227]; impacting the reproducibility and efficacy [228] and resulting in negative commercial implications for those looking to develop products.

Discussions with industry partners echo this challenge, noting that obtaining consistent bioactive profiles is already difficult and that regulatory pathways, particularly for nutritional claims, rely heavily on demonstrating reproducible activity and composition. The European Union has attempted to address this bottleneck for food products, adding 20 algae species to its novel food register in 2024, bringing the total to 60 [223]. Together, these challenges underscore the need for improved cultivation control, validated and accessible analytical pipelines, and harmonised standards to ensure reliable, batch-to-batch consistency of marine biomass and extracts or a need to shift product and business design to accommodate the changing extract profiles.

#### 5.1.2. Heavy Metals and Testing Burdens

As mentioned in Section 4.1, macroalgae are also prone to the accumulation of heavy metals and toxins and can been used as an indicator for environmental assessments [229]; similarly, microalgae can be used for bioremediation [230]. Whilst trace metals such as iron, cadmium, magnesium and iodine are important for human health [227], studies have shown that the levels of toxic metals (including aluminium, arsenic, cadmium and chromium) in some macroalgal species can easily exceed the WHO recommended guidelines [231]. The selection of a suitable macroalgal species and geographic location accounting for water quality and water flow rate [227] can substantially minimise the risks of heavy-metal accumulation, but this needs to be supported by regular batch testing. One company in the focus group shared that testing each harvest of macroalgae for pathogens and heavy metals as part of the sourcing requirements of their clients was cost-prohibitive and led them to stop certain product lines and exports. Annamalai and Kolandhasamy [3] also highlight a need to standardise the extraction and testing protocols themselves to support better data collection and replicability.

#### 5.1.3. Control Measures and Optimisation

The situation can be significantly different for microalgae, where controlled cultivation and strain selection markedly reduces variability [226]. Unlike many microorganisms, microalgae can be cultivated under semi-controlled conditions using open pond systems in large-scale volumes (>4000 m^3^), enabling reproducible biomass generation independent of seasonal variability and a high productivity rate in controlled closed systems such as photobioreactors.

Industrial cultivation systems also allow the optimisation of growth conditions to enhance the production of specific metabolites, supporting their translation into pharmaceutical, nutraceutical, and cosmeceutical products [7,11]. For example, the use of green light wavelengths improves the yield of phycobiliproteins from *Porphyridium purpureum*, whilst under multi-chromatic LED wavelengths the yields of other products such as eicosapentaenoic acid, *β*-carotene and exopolysaccharides are improved [12].

### 5.2. Manufacturing and Commercialisation

#### 5.2.1. Approaches to Commercial Production

Commercial macroalgae harvesting and production have expanded substantially in recent decades, driven by increasing demand for sustainable biomass across food, feed, biorefinery, environmental, and biotechnology sectors. Macroalgae, contributing approximately 28% of total global marine aquaculture output and generating an estimated USD 5 billion annually, represent one of the fastest-growing segments of the blue bio-economy [14]. Commercial systems in Europe still largely rely on wild harvesting [13,232], although globally 96.6% of algae is cultivated [15] in coastal farms, nearshore longline systems, and shallow-pond configurations tailored for high biomass yields and low energy inputs [14].

There is also long history of microalgae biomass exploitation, mainly for biofuel, food and feed applications, and in recent decades for pharmaceutical applications [233] however, most of those efforts were done using standard well-known commercial microalgae such as *Nannochloropsis*, *Chlorella* or *Limnospira* [234]. In recent years, several new species of microalgae that have been identified and scaled up to large-scale production in both open and closed systems are providing an entirely new source of bioactive compounds that can be extracted from the produced biomass. There are several examples including the dinoflagellate *Amphidinium carterae* [235,236], the cyanobacteria *Leptolyngbya* sp. [237,238], and the green microalgae *Monoraphidium* sp. [83,239].

Seasonal macroalgae harvests and batched microalgae production mean that large amounts of biomass must be processed quickly. In a UK context, there is a distinct lack of infrastructure and processing facilities as they rely on commercial-scale, food-grade machinery which has both a high upfront investment cost and a high operational cost (maintenance and cleaning burden). High-volume processing is only needed for a short period, so some processing sites work through community ownership models or on a rented access basis. Whilst there are still challenges around seasonal demand, in terms of access and the financial sustainability of the facilities themselves, this open innovation approach significantly reduces the barriers to entry for SMEs, boosts their innovation capacity and improves their technical capabilities, which can lead to higher-quality products and specialisation [233]. Additionally, there is a growing body of research focusing on developing and optimising processing approaches that are faster, cheaper and more energy efficient, whilst maintaining the extract quality, biological activity, stability and composition consistency needed for high-value applications [240].

#### 5.2.2. Regulatory Considerations

Feedback from the focus group highlighted manufacturing and regulatory burdens as significant barriers to commercialisation across high-value bioproduct classes (nutraceutical, cosmetic, pharmaceutical). Manufacturing complexity interacts with formulation needs, delivery routes, stability, and safety testing requirements, all of which must be addressed through rigorous, standardised quality frameworks. Translation is also slowed by a lack of marine-specific frameworks and safety data and, of course, the need for extensive toxicological, pharmacokinetic, and clinical validation [3].

The commercialisation of food products comes with its own regulatory constraints. In Europe, both macro- and microalgae are covered by a range of legislation, such as Novel Food Regulations (EU) 2015/2283 (authorization and safety assessment of novel foods), Feed and Food Hygiene Regulations and environmental regulations [241]. The focus group also highlighted the potential for certain regulations to have hidden impact and commented on the challenges of managing misalignment in international policies as SMEs. Taking iodine as an example, the European Union recently set an upper daily limit at 600 μg/day, five times lower than the level in Japan, which is set at 3000 μg/day [242]. This constrains product development and marketability, particularly of macroalgae in European markets, shifting business development decisions.

At a local level, environmental legislation, licencing and regulation tends to have the largest impact. Companies participating in the focus group expressed that guidance and licencing is generally welcomed, particularly amongst those sourcing from wild-harvested macroalgae as they want support to ensure they can scale sustainably. At the other end of the scale, policy developments related to the regulation of Digital Sequence Information (DSI) and the new high-seas governance framework under the Biodiversity Beyond National Jurisdiction (BBNJ) Agreement will directly affect how marine genetic resources, sequence data, and derivative bioactives collected from international waters can be accessed, shared, and commercialised with yet-untold impacts on businesses [243].

#### 5.2.3. Financing Research and Development

As in most sectors, the current investment landscape is very challenging, but translation is particularly difficult because of the substantial early capital needed to drive medical research. Industry professionals warned that long development timelines (up to 10 years and EUR 100M for new biomedicines) demand early “fail-fast” strategies and better screening tools to minimise costs and attrition. Supported pathways for the screening and identification of novel compounds can significantly help new product development, providing insights into the most promising compounds and the most appropriate species and growth conditions. Support is often sourced through public funding in collaboration with academia, a particularly important pathway for smaller companies.

The high resource commitment of medical research means several of the companies interviewed are taking a portfolio business model approach. Many are looking at animal nutrition as the fastest route to develop and commercialise higher value products, before moving onto human nutrition and later pharmaceuticals. Companies are also taking a circular approach from a materials point of view, creating a complementary portfolio of products that enable the use of the whole biomass with a particular focus on biostimulants (fertilisers) and residual fibre at the lowest tiers of functional and monetary value.

### 5.3. Value of Multidisciplinary Consortia in De-Risking Translation

**Figure 3:**
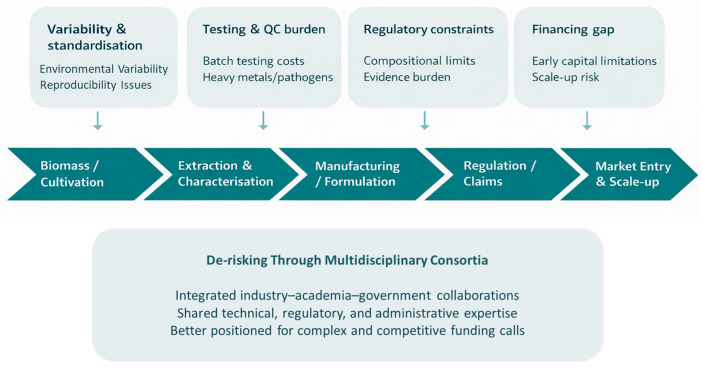
SME-identified translational bottlenecks in marine biodiscovery. Schematic overview of key challenges identified by companies across the marine biodiscovery pipeline, spanning biomass/cultivation, extraction and characterisation, manufacturing/formulation, regulatory approval, and market entry/scale-up. Four interrelated domains were highlighted: (1) variability and lack of standardisation in bioactive composition due to environmental influences, primarily affecting biomass production and early characterisation stages; (2) testing, quality control (QC), and manufacturing burden associated with batch-level safety and consistency requirements, impacting extraction through to formulation; (3) regulatory constraints linked to product classification, evidence requirements, and compositional limits, influencing formulation and approval stages; and (4) a financing and commercialisation gap, where limited early capital drives prioritisation of lower-barrier markets (e.g., animal nutrition) over longer, higher-risk therapeutic development, limiting progression from late-stage development to market entry. Multidisciplinary consortia (industry–academia–government) act as key enablers across all stages by providing shared expertise and improving competitiveness for complex funding and translational progression.

Collaboration between SMEs, academia, and government agencies (through the triple-helix model of innovation) is an important way to overcome technical and financial risks, building regional innovation ecosystems that advance progress towards business and policy goals [244,245,246]. The focus group discussed the highly competitive nature of the current funding landscape, with EU success rates at an average of 12% and some as low as 2% [247], making it difficult for SMEs to secure grants without strong consortia. Funding, at all scales, also comes with a high administrative burden, and some organisations can be unprepared for, and even surprised by, the project management resources needed to handle complex reporting and deliverables. Additionally, whilst the companies participating in the focus group generally felt comfortable applying for grants, they all had a deep appreciation for the time, complexity and skill needed to write successful applications. To this end some companies employ funding officers (with application writing included in their mandate), whilst many micro-SMEs turn to academics or external consultants to support in this capacity. An overview of the key translational stages, associated bottlenecks, and the role of multidisciplinary consortia in mitigating these risks is presented in Figure 3.

At the European level, several large-scale initiatives provide concrete examples of how multidisciplinary consortia de-risk marine biodiscovery. The EU-funded EMBRIC (European Marine Biological Research Infrastructure Cluster) brought together multiple European research infrastructures and industry stakeholders to establish integrated workflows for accessing biological, analytical, and data resources, while strengthening links between science and industry and facilitating technology transfer [248,249]. Similarly, the European Marine Biological Resource Centre (EMBRC) operates as a pan-European infrastructure spanning more than 80 marine institutes and participating in over 20 EU-funded projects, providing coordinated access to marine biodiversity, specialised facilities, and transnational research services that support both academic and industrial users [250,251]. Earlier collaborative programmes such as PharmaSea further demonstrated the value of interdisciplinary consortia by integrating academia and industry to address key bottlenecks in marine biodiscovery, including compound isolation, validation, and sustainable supply, ultimately reducing time to market [252,253]. More recently, Horizon Europe projects such as COMBO are addressing critical challenges in scalability and supply through synthetic biology, omics technologies, and advanced cultivation strategies, reinforcing the role of consortia as enablers of translation [254].

As mentioned in Section 5.2.3, supported pathways for compound screening and identification can have a transformative impact on product development and commercialisation. The Celtic Advances Life Science Innovation Network (CALIN) was a project funded through the European Regional Development Fund (ERDF) from 2016 to 2023. It brought together life sciences experts from three Welsh and three Irish Universities to create a supported R&D pipeline. The project supported more than 200 companies, facilitating access testing and expertise to test their products and ideas, and stimulating more than EUR 5 million of private investment to match public support [255]. Initiatives of this nature demonstrate how structured consortia can effectively de-risk early-stage innovation and accelerate translation.

The focus group also highlighted the importance of national and regional innovation support mechanisms that complement EU-level funding. Institutional programmes such as Enterprise Ireland, alongside the Welsh Government’s Smart Flexible Innovation Support (SFIS), have a strong track record in promoting industry–academia collaboration through targeted funding for feasibility studies and small-to-medium-scale projects (up to approximately GBP 200k) [246]. These schemes play a crucial role in bridging early-stage funding gaps, enabling SMEs to generate preliminary data, develop partnerships, and position themselves competitively for larger collaborative funding opportunities.

### 5.4. Future Directions for Marine Biodiscovery Across Oncology, Regeneration, and Skin Applications

Marine-derived compounds are gaining global traction; with around 20 marine-derived natural products currently approved for medicinal use [256,257], the past 10 years have seen a slow but steady increase in the number of approved products with many more natural products undergoing clinical trials [258,259]. However, this is a small drop in the ocean compared to the vast potential of natural products in the marine environment, highlighted by the high success rate of marine natural products; for example, Almaliti and Gerwick [257] report that 23 marine-inspired agents are in clinical use from a total of 39,238 marine natural products, a success rate nearly nine times better than the industry standard of 15,000:1. This is unsurprising when we consider the vast biodiversity of the ocean, which contains 34 of the ~35 recognised phyla, compared to the 15 phyla represented in terrestrial environments [260]; however, this scale and variety also constrains the field and reaffirms the need for coordinated translational infrastructures [256]. Integrating scalable cultivation platforms, predictive preclinical systems (e.g., organoids, advanced skin equivalents), and coordinated funding mechanisms will be critical to enabling these marine bioactives to transition successfully into therapeutics, biomaterials, and next-generation skincare products [258,259].

## 6. Conclusions

Marine biodiscovery is emerging as a versatile and sustainable platform for biotechnology, supported by the exceptional chemical and functional diversity of marine microalgae and macroalgae. These organisms inhabit dynamic and often extreme environments, which drives production of structurally diverse metabolites involved in ecological defence, signalling and stress adaptation. This biochemical diversity creates opportunities across nutraceutical, cosmetic, regenerative, and anticancer applications. As a sustainable and scalable source of novel bioactive compounds, algae have strong potential to contribute future innovation in health and biotechnology.

A key message of this review is that marine biodiscovery must be aligned with clearly defined translational goals. Application-driven approaches require robust molecular characterisation, mechanistic understanding, and careful evaluation of structure–activity relationships. This is particularly important for compound classes such as fucoidan, ulvan, phlorotannins, and carotenoids, where biological activity is highly dependent on molecular weight distribution, sulphation pattern, degree of polymerisation, stereochemistry and formulation stability. Future progress will therefore depend on integrated frameworks combining controlled cultivation, scalable bioprocessing, validated analytics, and application-specific testing models.

Advances in large-scale microalgal cultivation now support reproducible biomass production and enable metabolite profiles to be tuned under defined culture conditions. In parallel, omics technologies, metabolite fingerprinting, and high-content phenotypic screening are improving the identification of lead compounds and the mapping of their mechanisms of action. Physiologically relevant in vitro and ex vivo models, including 3D skin, organoids, and cancer spheroids, offer further opportunities to assess efficacy, toxicity, and delivery in systems that better reflect human biology. Alongside these scientific advances, environmental sustainability must remain central, ensuring that algal cultivation and harvesting support secure supply chains while protecting marine ecosystems and coastal communities.

Insights from Welsh and Irish SMEs further highlight that scientific innovation must be matched by practical routes to translation. Key barriers include biomass variability, heavy-metal accumulation, processing infrastructure, regulatory hurdles, and the high cost of early-stage research. Collaborative innovation ecosystems linking business, academia, and government will be essential to de-risking early-discovery research, supporting validation and accelerating progression towards regulated markets. This is particularly important for SMEs, where targeted funding and integrated partnerships can help bridge the gap between ecological discovery, preclinical development, and commercial application.

Overall, marine biodiscovery is well positioned to contribute to sustainable health innovation. By aligning discovery with translational need, applying advanced cultivation and analytical technologies, and building collaborative, application-focused research pipelines, algae-derived bioactives can support the development of next-generation products across human health, biotechnology, and the circular bio-economy.

## Figures and Tables

**Table 2:** Representative cytotoxic and anti-proliferative activities of marine-derived bioactive compounds. The up arrow (↑) indicates an increasing change.

Functional Class	Compound	Representative Chemical Structure	Source Species	Cell Line/Model	Assay/IC50 (Units)	Mechanism(s)	Key Ref.
Alkaloids (bis-indole)	Caulerpin	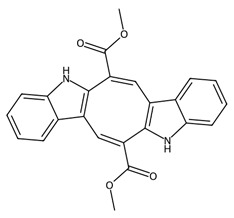	*Sargassum platycarpum*	HepG2 liver cancer	Cell viability assay; IC50 24.6 ± 2.1 µg/mL	Cytotoxicity reported; mechanistic attribution limited (computational nucleobase interaction explored)	[131]
Macroalgal extract (multi-class mixture)	80% ethanol extract (CSE; contains caulerpin and polyphenols by HPLC-MS)	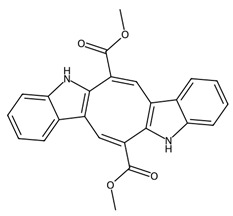	*Caulerpa sertularioides*	SKLU-1 lung adenocarcinoma (2D vs. 3D)	Viability (Sytox Green), 24 h: IC50 80.28 µg/mL (2D) vs. 530 µg/mL (3D)	Intrinsic and extrinsic apoptosis; caspase-3/7 up; ΔΨm loss; S and G_2_/M arrest; reduced invasion in 3D	[132]
Microalgal extract (complex mixture)	Biomass extracts (nutrient-replete vs. nutrient-stressed conditions)	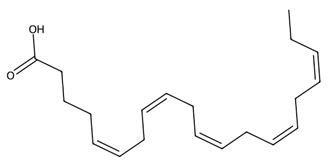 e.g., EPA (20:5 n-3)	*Porphyridium purpureum; Nannochloropsis oculata*	SKOV3 ovarian cancer (2D and 3D models; migration assays)	Cell viability assays (RT-glow) Live/dead fluorescence assay (3D spheroids, 72 h) Scratch wound migration assay	Anti-proliferative and anti-migratory activity; enhanced activity under nutrient-stressed cultivation; effects associated with changes in biomass composition and context-dependent responses between 2D and 3D models	[41]
	Crude ethanol extract (EEC)	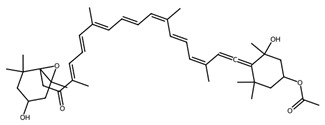	*Chaetoceros calcitrans* (diatom; microalga)	MCF-7 breast vs. MCF-10A non-tumoral breast	IC50 3.00 ± 0.65 µg/mL (MCF-7, 24 h)	Apoptosis without cell-cycle arrest; Bax/Bcl-2 ratio ↑; caspase-7 pathway; regulation of CDK2/MDM2/p21/cyclins	[133]
Phenolics (phlorotannin)	Dieckol	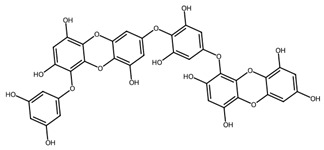	*Ecklonia cava*	SKOV3 and A2780 ovarian cancer cell lines and SKOV3 xenograft models	MTT; IC50 84.3 μg/mL (A2780) and 99.6 μg/mL (SKOV3). Annexin V/PI staining (apoptosis) Xenograft model: tumour weight inhibition ~21.2–41.8% at 50–100 mg/kg	ROS generation; mitochondrial apoptosis; activation of caspase-8, -9, and -3; apoptosis confirmed by Annexin V/PI; tumour growth suppression in vivo	[129]
Pigments/lipids (carotenoid)	Fucoxanthin (pure standard; compared to fucoxanthin-rich extracts)	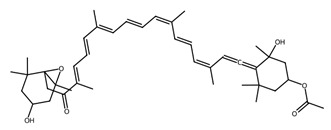	*Undaria pinnatifida*	Multiple cancer lines (incl. MCF-7 breast; A549 lung; Lovo colon; Malme-3M melanoma)	MTT; examples at 72 h: MCF-7 22.48 ± 1.26 µM; A549 25.57 ± 1.07 µM; Lovo 21.83 ± 1.17 µM; Malme-3M 17.33 ± 2.65 µM	Growth inhibition; time- and dose-dependence; mechanisms discussed include apoptosis and cell-cycle effects (model-dependent)	[126]
	Fucoxanthin	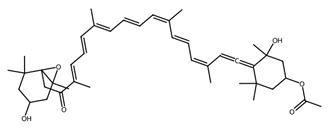	Brown Algal carotenoid	HEC-1A endometrial cancer	MTT; IC50 7.5 µM	ROS elevation, mitochondrial dysfunction; Bax/caspase-3 up; Bcl-2/cyclin D1 down; PI3K/Akt/mTOR inhibition	[128]
Polyketides	Okadaic acid	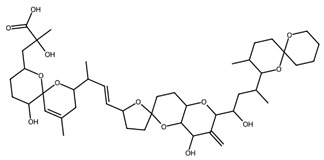	Dinoflagellates (e.g., *Prorocentrum*/*Dinophysis* producers)	U-937 leukaemia; MG63 osteosarcoma	Cytotoxicity assays; IC50 100 nM (U-937) and 75 nM (MG63)	PP2A/PP1 inhibitor (toxin class); ROS/MAPK-mediated mitochondrial caspase-dependent death (U-937); PKR/NF-κB/caspase involvement (MG63)	[129]
Polysaccharides	Ulvan sulphated hetero-polysaccharide	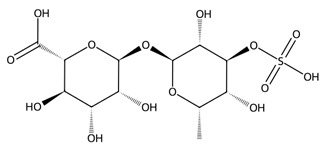 Ulvanobiouronic acid (ulvan repeat)	*Ulva lactuca*	Hepatocellular carcinoma; breast; cervical (three human cancer lines)	Cytotoxicity assay; IC50 29.67 ± 2.87 µg/mL (HCC), 25.09 ± 1.36 µg/mL (breast), 36.33 ± 3.84 µg/mL (cervical)	Mechanism not resolved in abstract; structural composition and sulphation reported	[130]
	Fucoidan (low-kDa range reported)	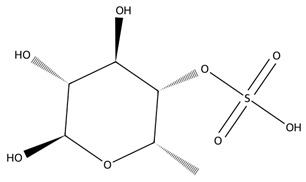 α-L-fucose-4-sulphate (fucoidan repeat)	*Stoechospermum marginatum*	HepG2 liver cancer; Vero (normal)	MTT; IC50 24.4 ± 1.5 µg/mL (HepG2); null cytotoxicity on Vero	Apoptosis/necrosis-like phenotypes reported (AO/EB); DNA fragmentation consistent with necrotic death under stated conditions	[134]
	Laminarin (low-kDa range reported)	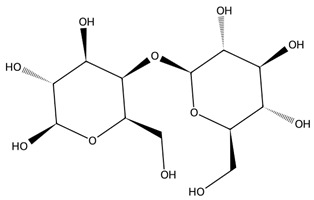 Laminaribiose (laminarin repeat)	*Padina pavonica*	HT-29 colon cancer; Vero (normal)	MTT; IC50 57 ± 1.2 µg/mL (HT-29); null cytotoxicity on Vero	Apoptosis/necrosis-like phenotypes reported (AO/EB)	[134]
Terpenes (halogenated sesquiterpenes)	(−)-Elatol	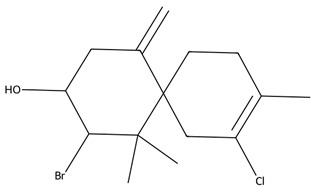	*Laurencia dendroidea*	Colo-205 colon cancer	Cytotoxicity assay; IC50 2.5 ± 1.3 µg/mL	Apoptosis induction: caspases 2/4/6/8 implicated	[88]
	Obtusol	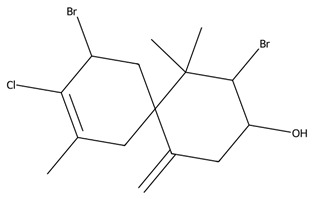	*Laurencia dendroidea*	Colo-205 colon cancer	Cytotoxicity assay; IC50 1.2 ± 1.4 µg/mL	Apoptosis induction: caspase-6 implicated	[88]

## Data Availability

No new data were created or analyzed in this study. Data sharing is not applicable to this article.

## Abbreviations

The following abbreviations are used in this manuscript:

Akt	Protein Kinase B
AP-1	Activator Protein-1
BBNJ	Biodiversity Beyond National Jurisdiction
BMDM	Bone Marrow-Derived Macrophage
BMPs	Bone Morphogenic Proteins
CAGR	Compound Annual Growth Rate
CDK	Cyclin-Dependent Kinase
COX-2	Cyclooxygenase-2
CXCR4	C-X-C Motif Chemokine Receptor 4
DGDG	Digalactosyldiacylglyceride
DGLA	Dihomo-γ-Linolenic Acid
DSI	Digital Sequence Information
ECM	Extracellular Matrix
EGFR	Epidermal Growth Factor Receptor
EMT	Epithelial–Mesenchymal Transition
EPA	Eicosapentaenoic Acid
ERDF	European Regional Development Fund
ERK	Extracellular Signal-Regulated Kinase
EU	European Union
FTIR	Fourier Transform Infrared Spectroscopy
FSA	Food Standards Agency
GPx	Glutathione Peroxidase
HCC	Hepatocellular Carcinoma
HIF-1α	Hypoxia-Inducible Factor 1-Alpha
HO-1	Heme Oxygenase-1
HPLC-MS	High-Performance Liquid Chromatography–Mass Spectrometry
IC_50_	Half-Maximal Inhibitory Concentration
IFN-γ	Interferon-Gamma
IGF-1R	Insulin-Like Growth Factor-1 Receptor
IL-1β/IL-6	Interleukin-1 Beta/Interleukin-6
iNOS	Inducible Nitric Oxide Synthase
JAK-STAT	Janus Kinase/Signal Transducer and Activator of Transcription
JNK	c-Jun N-terminal Kinase
LMF	Low-Molecular-Weight Fucoidan
LPS	Lipopolysaccharide
MAAs	Mycosporine-Like Amino Acids
MAPK/MAPK(s)	Mitogen-Activated Protein Kinase(s)
MGDG	Monogalactosyldiacylglyceride
MMPs	Matrix Metalloproteinases
MSCs	Mesenchymal Stem Cells
mTOR	Mammalian Target of Rapamycin
NAFLD	Non-Alcoholic Fatty Liver Disease
NF-κB	Nuclear Factor Kappa-B
NQO1	NAD(P)H Quinone Dehydrogenase 1
NO	Nitric Oxide
NOX2	NADPH Oxidase 2
NSCLC	Non-Small-Cell Lung Cancer
OA	Osteo Arthritis
PGE_2_	Prostaglandin E_2_
PI3K	Phosphoinositide 3-Kinase
PM	Particulate Matter
PUFA(s)	Polyunsaturated Fatty Acid(s)
RCT	Randomised Controlled Trial
ROS	Reactive Oxygen Species
SFIS	Smart Flexible Innovation Support
SME(s)	Small and Medium-Sized Enterprise(s)
SQDG	Sulfoquinovosyldiacylglyceride
TAGs	Triacylglycerols
TGF	Transforming Growth Factor
TGF-β	Transforming Growth Factor Beta
TNF-α	Tumour Necrosis Factor-Alpha
TYR	Tyrosinase
VEGF/VEGFR2	Vascular Endothelial Growth Factor/Receptor 2
WT	Wild-Type

## Appendix A

**Table A1:** International clinical trials using micro and macro algae extracts for nutrition, oncology, regenerative medicine and skincare applications.

Therapeutic Category	Trial Name/Acronym	Algae Source	Active Compound/Derivative	Trial ID	Status	Phase	Year	Location	Key Ref(s)
Cardiovascular/Gut Health	CALGUT—Spirulina and Gelidium corneum on cardiovascular risk and gut microbiome	*Arthrospira platensis* (microalga) and *Gelidium corneum* (red macroalga)	Spirulina biomass; *Gelidium corneum* extract	NCT07173062	Recruiting	N/A	2025–2026	Portugal	[261]
Gut Health	Himanthalia elongata brown seaweed on gut microbiota in overweight adults	*Himanthalia elongata* (brown macroalga)	Whole biomass (encapsulated)	Published 2025—Nutrients	Completed	RCT	2024–2025	Spain	[262]
	Carrageenan food additive and the human gut microbiome	Red macroalgae (carrageenan-producing species)	Carrageenan (sulphated polysaccharide)	NCT06738329	Recruiting	N/A	2024–2025	UK	[263]
	Tetrasol microalgae extract on gut health, anxiety and immune function	*Tetraselmis* sp. (microalgae)	Tetrasol extract	NCT06425094	Completed	Phase I	2024–2025	USA	[264]
Inflammation	Spirulina protective/regenerative effects in hepatectomy liver injury	*Arthrospira platensis* (Spirulina, microalgae)	Whole biomass	NCT07263217	Recruiting	N/A	2025–2028	China	[265]
	ESSAY—Spirulina supplement for benign thyroid nodules	*Arthrospira platensis* (Spirulina, microalga)	Whole biomass	NCT03535974	Completed	N/A	2018–2020	Romania	[266]
	Fucoidan supplement for fatigue and inflammation in cancer survivors	*Fucus vesiculosus* and *Undaria pinnatifida* (brown macroalgae)	Fucoidan	NCT06295588	Recruiting	Phase II	2025–2026	USA	[208]
Metabolic/Obesity	Spirulina platensis + calorie restriction in obese men	*Arthrospira platensis* (Spirulina, microalgae)	Whole biomass	NCT06076161	Completed	N/A	2023–2024	Indonesia	[267]
	Spirulina supplementation in overweight/obese adults	*Arthrospira platensis* (Spirulina, microalgae)	Whole biomass	NCT02993627	Completed	N/A	2017–2019	Iran	[268]
	ALGAENERGY—Spirulina and Chlorella on metabolic syndrome biomarkers	*Arthrospira platensis* and *Chlorella vulgaris* (microalgae)	Whole biomass	NCT05343858	Completed	N/A	2022–2023	Spain	[269]
	Algae-oil-fortified soymilk vs. capsules—EPA/DHA bioavailability	Unknown	EPA + DHA	NCT05802797	Active	Early Phase I	2023–2026	USA	[270]
Nutrition	Spirulina as daily nutritional supplement in Cambodian pre-school children	*Arthrospira platensis* (Spirulina, microalgae)	Whole biomass	Barennes et al. 2022	Completed	RCT (crossover)	2019–2021	Cambodia/France	[271]
	NovAL—nutrient bioavailability from Chlorella and Nannochloropsis	*Chlorella pyrenoidosa + Nannochloropsis salina* (microalgae)	Whole-biomass micronutrients (iron, iodine, carotenoids, B12)	NCT04567823	Completed	N/A	2021–2022	Germany	[272]
	ALG—algae oil immune response and lipid bioavailability	*Schizochytrium* sp. (Microalage, including strain FCC3204)	DHA (Origins™ 550-Y (Fermentalg) and Oleo H-02 (Microalgas))	NCT07086573	Upcoming	N/A	2026	Netherlands	[273]
	GOBO 2—bioavailability of EPA + DHA from two microalgal sources vs. fish oil	*Schizochytrium* sp. (microalgae)	DHA + EPA (life’s™ Omega O1035DS nTG)	NCT07241377	Recruiting	Phase III	2025–2026	Australia	[274]
	GOBO—bioavailability of EPA + DHA: microalgal vs. fish oil	*Schizochytrium* sp. (microalgae)	DHA + EPA (life’s™ Omega O1035DS)	NCT06629103	Active	Phase IV	2024–2025	Australia	[275]
	DHA from algae supplement in vegetarians	*Schizochytrium* sp. (microalgae)	DHA-O	NCT04278482	Completed	N/A	2020–2021	Spain	[276]
	Algae-oil-fortified soymilk vs. capsules—EPA/DHA bioavailability	Unknown	EPA + DHA	NCT05802797	Active	Early Phase I	2023–2026	USA	[270]
Oncology	Fucoidan safety and efficacy in squamous cell carcinoma of the head and neck	Brown macroalgae (fucoidan-producing species)	Fucoidan (MW ~20,000 Da)	NCT04597476	Unknown	Phase II	2020–ongoing	Taiwan	[207]
	Auxiliary effects of fucoidan for locally advanced rectal cancer patients (neoadjuvant CCRT)	Brown macroalgae (fucoidan-producing species)	Fucoidan	NCT04342949	Recruiting	N/A	2018–ongoing	Taiwan	[210]
	Oligo-fucoidan in advanced hepatocellular carcinoma (HCC)	Brown macroalgae (fucoidan-producing species)	Oligo-fucoidan (MW 500–800 Da)	NCT04066660	Terminated	N/A	2019–2022	Taiwan	[211]
	Oligo-fucoidan QoL adjuvant in NSCLC patients receiving platinum-based chemotherapy	Brown macroalgae (fucoidan-producing species)	Oligo-fucoidan	NCT03130829	Withdrawn	N/A	Withdrawn—insufficient target population	Taiwan	[212]
	Kahalalide F clinical development programme	*Bryopsis* sp. (green macroalgae)	Kahalalide F (cyclic depsipeptide)	Multiple (5 trials)	Terminated	Phase I/II	2001–2010	Spain	[213]
	Elisidepsin (Irvalec^®^/PM02734) + erlotinib in advanced solid tumours	*Bryopsis* sp. (green macroalga)—synthetic kahalalide F derivative	Elisidepsin/PM02734 (Irvalec^®^)—synthetic cyclodepsipeptide	NCT00884845	Terminated	Phase I	2009–2012 (clinical development discontinued)	Spain; multinational EU	[215]
	Monoclonal gammopathy of undetermined significance and smouldering multiple myeloma (NUTRIVENTION-3)	Microalgae (unknown sp.)	Algae-derived omega-3 fatty acids (DHA/EPA) combined with curcumin	NCT05640843	Recruiting	N/A	2022-2026	USA	[277]
	Brentuximab Vedotin (Adcetris^®^)	*Symploca* sp./*Lyngbya majuscula* (Cyanobacteria/microalga)	Monomethyl auristatin E (MMAE)—dolastatin 10 analogue	ADCETRIS/BV (FDA Approved 2011)	Approved	Approved	2009–2011 (approval)	USA	[278]
Oncology/Inflammation	Phycocyanin (Spirulina-derived) vs. chemotherapy-induced peripheral neuropathy in metastatic gastric cancer	*Arthrospira platensis* (Spirulina, microalga)	Phycocyanin (biliprotein pigment)	NCT05025826	Recruiting	N/A	2021–ongoing	China	[216]
	Fucoidan for preventing chemotherapy-related fatigue in GI/GYN cancer (Mayo Clinic)	Brown macroalgae (fucoidan-producing species)	Oligo-fucoidan	NCT06855524	Recruiting	Phase II	2025–ongoing	USA	[209]
Regenerative Medicine	Alginate-encapsulated human islet cell implants in peritoneal cavity—pilot in Type 1 diabetes	Brown macroalgae (alginate-producing species)	Alginate microcapsule-encapsulated human islet cells	NCT01379729	Completed	N/A (Pilot)	~2011–2013	Belgium	[279]
	DIABECELL^®^ Phase I/IIa—alginate-encapsulated porcine islets xenotransplantation in Type 1 diabetes	Brown macroalgae (alginate-producing species)	Alginate microencapsulation of porcine islets (DIABECELL^®^ device)	NCT00940173	Completed	Phase I/II	2009–2012	New Zealand	[280]
	DIABECELL^®^ Phase IIb—alginate-encapsulated porcine islets safety and efficacy in Type 1 diabetes	Brown macroalgae (alginate-producing species)	Alginate microencapsulation of porcine islets (DIABECELL^®^ device)	NCT01736228	Completed	Phase II	2012–2017	New Zealand/Russia	[281]
	Beta-Air bioartificial pancreas (macro-encapsulated islets) implantation in Type 1 diabetes	Brown macroalgae (alginate-producing species)	Alginate hydrogel macro-encapsulation device (Beta-Air) containing human pancreatic islets	NCT02064309	Active	Phase I/II	2014–ongoing	Sweden/Israel	[282]
	Fucoidan (galactofucan sulphate) oral ingestion and haematopoietic progenitor cell (CD34+) mobilisation	Brown macroalgae (fucoidan-producing species)	Galactofucan sulphate/fucoidan	Irhimeh et al. 2007 (PubMed 17533053)	Completed	Pilot clinical trial	~2005–2006 (published 2007)	Australia	[283]
Skincare	Dunaliella bardawil (9-cis-β-carotene) adjuvant to NB-UVB phototherapy in plaque psoriasis	*Dunaliella bardawil* (green microalga)	9-cis-β-carotene-rich Dunaliella bardawil powder	NCT01628081	Unknown	Phase III	2012–unknown	Israel	[284]
	Gracilaria algae 3% topical cream vs. clobetasol 0.05% in plaque psoriasis	*Gracilaria* sp. (red macroalga)	Phlorotannins/anti-inflammatory polyphenols	Shatalebi et al. 2020 (PubMed 32951292)	Completed	N/A	~2018–2019 (published 2020)	Iran	[285]
	SXRG84 (*Ulva* sp. sulphated polysaccharide) in inflammatory skin conditions—Bio-Belly 2 sub-study	*Ulva* sp. (green macroalga)	Sulphated xylorhamnoglucuronan (SXRG84)	Roach et al. 2023	Completed	N/A	~2021–2022 (published 2023)	Australia	[286]
	PCA—Algae formulations (UMAC and golden-brown algae) on psoriasis skin lesions, lipids and inflammation	Unique Marine Algae Concentrate (UMAC) and Golden-brown algae (microalgae blend)	UMAC whole-cell concentrate; whole biomass	NCT01045395	Completed	Phase I	2009–2012	Canada	[287]
